# Association Between Postoperative Mechanical Alignment and Clinical Outcomes After Medial Opening-Wedge High Tibial Osteotomy: A Retrospective Cohort Study

**DOI:** 10.3390/jcm15208110

**Published:** 2026-10-19

**Authors:** Muhammed Kazez, Ömer Cihan Batur, Gökhan Önce, Oğuz Kaya, Oğuz Aydın, Hacı Bayram Tosun

**Affiliations:** Department of Orthopedics and Traumatology, Elazığ Fethi Sekin City Hospital, 23280 Elazığ, Türkiye; istanbultip@gmail.com (Ö.C.B.); gokhanonce@gmail.com (G.Ö.); oguzkayamd@gmail.com (O.K.); droguzaydin60@gmail.com (O.A.); bayramtosun@hotmail.com (H.B.T.)

**Keywords:** medial opening-wedge high tibial osteotomy, hip–knee–ankle angle, mechanical alignment, valgus correction, knee osteoarthritis, clinical outcomes

## Abstract

**Background:** The optimal coronal mechanical alignment after medial opening-wedge high tibial osteotomy (MOWHTO) remains controversial. This study aimed to evaluate the association between postoperative hip–knee–ankle angle (HKA) and early- to mid-term clinical outcomes and to explore whether this relationship showed evidence of nonlinearity within the observed valgus range. **Methods:** This single-center retrospective cohort study included 188 patients who underwent MOWHTO with a 10- or 12.5-mm osteotomy opening. The primary exposure was HKA measured at 6 weeks postoperatively, and the final follow-up International Knee Documentation Committee (IKDC) score was designated as the primary clinical outcome. Lysholm and visual analog scale (VAS) scores were evaluated as secondary outcomes. Associations between postoperative HKA and clinical outcomes were assessed using multivariable linear regression with HC3 heteroskedasticity-robust standard errors, while potential nonlinear relationships were explored by incorporating a quadratic HKA term. **Results:** The mean age was 52.64 ± 4.74 years, the mean follow-up was 25.48 ± 6.80 months, and the mean HKA at 6-weeks postoperatively was 2.88 ± 1.21° of valgus. IKDC and Lysholm scores significantly improved, whereas VAS scores decreased (all *p* < 0.001). In multivariable analyses, each 1° increase in postoperative valgus HKA was associated with a 1.49-point-higher final IKDC score (95% CI, 0.04–2.93; *p* = 0.044) and a 2.61-point-higher final Lysholm score (95% CI, 1.10–4.13; *p* < 0.001). No statistically significant association was observed with final VAS (β = 0.004; 95% CI, −0.050 to 0.058; *p* = 0.883). The quadratic HKA term was not statistically significant for any clinical outcome, indicating no statistically detectable nonlinearity within the observed 0.4–4.5° valgus range. The IKDC association was attenuated in an influence sensitivity analysis, whereas the Lysholm association remained significant. **Conclusions:** Within the restricted postoperative HKA range observed in this cohort (0.4–4.5° of valgus), greater postoperative valgus HKA was associated with higher functional outcome scores, with a more pronounced and consistent association for Lysholm and a more modest association for the primary outcome, IKDC. No association was observed with pain, and no statistically detectable nonlinearity was identified within the observed HKA range. These observational findings do not establish a causal valgus dose–response relationship, a universal optimal postoperative HKA, or the superiority of individualized correction. Prospective studies across broader alignment ranges are needed to define clinically appropriate alignment targets for specific patient subgroups.

## 1. Introduction

Medial compartment knee osteoarthritis associated with varus malalignment is an important cause of pain and functional impairment, particularly in active middle-aged patients. Medial opening-wedge high tibial osteotomy (MOWHTO) is a joint-preserving surgical procedure that aims to realign the mechanical axis of the lower extremity, redistribute load from the degenerative medial compartment toward the lateral compartment, and improve knee function in appropriately selected patients [1,2,3]. However, there remains no clear consensus regarding the optimal postoperative coronal alignment after MOWHTO [2,4].

The traditional correction strategy aims to shift the weight-bearing line toward the Fujisawa point, located at approximately 62.5% of the tibial plateau width [4,5]. However, more recent evidence suggests that this degree of valgus correction may not be necessary for every patient and that more conservative targets closer to neutral alignment may also provide satisfactory clinical outcomes [6,7]. While insufficient correction may result in persistent medial compartment loading and suboptimal clinical outcomes, excessive valgus correction may adversely affect clinical outcomes and increase mechanical stress on the lateral and patellofemoral compartments [6,8,9]. Accordingly, increasing attention has been directed toward individualized alignment strategies that account for patient-specific characteristics and the structural condition of the knee rather than applying a uniform correction target to all patients [10,11].

Coronal mechanical alignment after MOWHTO is commonly characterized using radiographic parameters such as the hip–knee–ankle angle (HKA) and medial proximal tibial angle (MPTA) [1,3,12,13]. Although postoperative alignment has been associated with patient-reported outcomes, the relationship between achieved postoperative HKA and early- to mid-term clinical outcomes remains incompletely defined, particularly regarding whether this association is linear across the postoperative valgus range [12,13]. Interpretation across studies is further complicated by heterogeneity in patient characteristics, intra-articular pathology, correction strategies, and surgical techniques [4,12].

Therefore, the primary aim of this study was to evaluate the association between postoperative HKA after MOWHTO and early- to mid-term knee function, using the final follow-up International Knee Documentation Committee (IKDC) score as the primary clinical outcome. Lysholm and visual analog scale (VAS) scores were evaluated as secondary clinical outcomes. We additionally explored whether the HKA–outcome relationship showed evidence of nonlinearity within the observed postoperative valgus range and assessed maintenance of mechanical correction during follow-up. We hypothesized that postoperative HKA within the observed valgus range would be associated with early- to mid-term functional outcomes.

## 2. Materials and Methods

### 2.1. Study Design and Ethical Approval

This retrospective cohort study included patients who underwent medial opening-wedge high tibial osteotomy (MOWHTO) at the Department of Orthopaedics and Traumatology, Elazığ Fethi Sekin City Hospital, between August 2021 and December 2025. The study protocol was approved by the Non-Interventional Research Ethics Committee of Elazığ Fethi Sekin City Hospital (21 May 2026; Decision No. 2026/30-26) and was conducted in accordance with the Declaration of Helsinki. Reporting followed the Strengthening the Reporting of Observational Studies in Epidemiology (STROBE) recommendations.

### 2.2. Patient Selection

All consecutive patients who underwent MOWHTO during the study period were assessed according to predefined eligibility criteria.

*Inclusion Criteria*

Patients were eligible for inclusion if they met all of the following criteria:

- Age between 35 and 65 years;
- Body mass index (BMI) ≤ 35 kg/m^2^;
- Symptomatic medial compartment knee osteoarthritis despite conservative treatment;
- Tibial-based varus deformity amenable to correction with isolated MOWHTO;
- Lateral distal femoral angle (LDFA) between 85° and 90°, with no clinically relevant femoral coronal deformity;
- Chondral and/or meniscal pathology involving the medial compartment on preoperative magnetic resonance imaging (MRI);
- Treatment using a TomoFix-type medial proximal tibial locking plate system (Trumed Medical^®^, İstanbul, Türkiye) with an osteotomy opening of either 10 or 12.5 mm; and
- A minimum clinical and radiographic follow-up of 12 months.

*Exclusion Criteria*

Patients were excluded if any of the following criteria were present:

- Inflammatory arthritis;
- Advanced lateral or patellofemoral compartment osteoarthritis;
- Previous high tibial osteotomy or other corrective osteotomy of the index knee;
- Concomitant anterior or posterior cruciate ligament reconstruction;
- History of traumatic or pathological fracture of the affected extremity;
- Malignancy, active infection, or sequelae of previous infection;
- Neuromuscular disease;
- Use of a fixation system other than the study implant or graft material other than an ipsilateral tricortical iliac crest autograft; or
- Missing clinical, radiographic, or preoperative data required for the primary analyses.

A total of 315 consecutive patients were assessed for eligibility, of whom 127 were excluded: incomplete postoperative radiographs (n = 3), unavailable clinical scores (n = 16), incomplete preoperative data (n = 12), an 8.5-mm osteotomy opening (n = 14), a 15-mm osteotomy opening (n = 11), follow-up <12 months (n = 10), surgery performed by a surgical team other than the study team (n = 14), use of a different implant system (n = 17), and use of a synthetic bone graft, allograft, or graft material other than ipsilateral tricortical iliac crest autograft (n = 30). Accordingly, 188 patients were included in the final analytical cohort.

Each patient contributed only one index knee to the analysis. Patients who had undergone MOWHTO of the contralateral knee before the study period contributed only the knee treated during the August 2021–December 2025 study period that fulfilled the study eligibility criteria. Thus, the final analytical cohort comprised 188 patients and 188 index knees, with no bilateral observations from the same patient. The patient-selection process and reasons for exclusion are presented in Figure 1.

### 2.3. Preoperative Planning, Alignment, and Selective Grafting Strategy

Preoperative assessment included standing weight-bearing full-length lower-extremity radiographs, knee magnetic resonance imaging (MRI), and clinical evaluation. Coronal mechanical-axis planning was performed on the picture archiving and communication system (PACS) using the Miniaci method [14,15], which enables calculation of the correction required to shift the weight-bearing axis toward a predefined reference on full-length weight-bearing radiographs.

Two mechanical-axis reference directions were defined during preoperative planning. The neutral mechanical axis was defined as a weight-bearing line passing through the center of the knee, approximately between the tibial eminences, corresponding to an HKA of approximately 0°. The Fujisawa reference was defined as a weight-bearing line passing just lateral to the lateral tibial eminence, corresponding to approximately 62.5% of the tibial plateau width measured from medial to lateral [16,17]. Examples of Miniaci planning toward these two reference directions are shown in Figure 2.

The final correction direction was determined using a predefined clinical decision algorithm that incorporated age, Kellgren–Lawrence (KL) grade, medial-compartment chondral damage, and medial meniscal pathology. Preoperative assessment included radiographs and MRI, whereas the medial-compartment chondral grade and meniscal pathology used in the correction algorithm were determined during diagnostic arthroscopy. These arthroscopic findings were combined with age and KL grade to complete the correction score before osteotomy opening and fixation.

One point was assigned for each of the following criteria: age < 55 years, KL grade 3, medial-compartment International Cartilage Repair Society (ICRS) grade 4, and the presence of a medial meniscus posterior root tear (MMPRT) or repairable medial longitudinal/bucket-handle tear. Patients with a total score of 0–1 were directed toward the neutral/slight-valgus reference, whereas those with a score of 2–4 were directed toward the Fujisawa reference. Individual scores of 0–4 were not mapped to separate fixed HKA values or weight-bearing-line (WBL) percentages; rather, the score was used categorically to select one of the two reference directions.

Once the correction direction had been selected, the required correction was assessed using the Miniaci planning framework, and patients included in the present cohort were treated using 10- or 12.5-mm osteotomy openings. Patient-specific final planned HKA values and WBL percentages were not systematically recorded as discrete numerical variables in the retrospective source records. Therefore, exact patient-level planned-versus-achieved alignment could not be reconstructed reliably. Postoperative 6-week HKA was consequently used as the measured achieved alignment for the primary analyses. The individualized correction-direction and selective-grafting algorithm is summarized in Table 1.

After completion of the correction score, the osteotomy was gradually opened and coronal mechanical alignment was verified intraoperatively under fluoroscopic guidance using the electrocautery-cord cable technique. The cable was aligned with the center of the ankle distally and the center of the femoral head proximally. With the knee positioned in a true anteroposterior projection, the point at which the mechanical-axis line crossed the tibial plateau was assessed fluoroscopically. Correction was directed toward the neutral/slight-valgus reference in patients with a score of 0–1 and toward the Fujisawa reference in those with a score of 2–4.

Bone grafting was determined independently of the correction score according to osteotomy gap width and lateral cortical-hinge integrity. Grafting was not routinely performed for a 10-mm opening with an intact lateral hinge, whereas graft requirement was reassessed for a 12.5-mm opening; a 12.5-mm opening alone was not considered an absolute indication for grafting. Lateral-hinge disruption favored graft use at either opening size. This selective strategy was based on the recognized influence of opening-gap magnitude and lateral-hinge integrity on osteotomy stability and healing [18,19]. When grafting was considered necessary, an autologous tricortical graft harvested from the ipsilateral anterior iliac crest was used.

### 2.4. Surgical Technique

All procedures were performed by the same six-member orthopedic surgical team experienced in knee surgery, with three surgeons serving as the primary operating surgeons. Throughout the August 2021–December 2025 study period, there were no deliberate changes in the standardized MOWHTO surgical technique, Miniaci-based planning method, TomoFix-type locking plate system, osteotomy approach, or clinical decision protocol used to determine the correction direction. Spinal or general anesthesia was administered according to clinical and anesthesiologic assessment. Approximately 30 min before skin incision, intravenous cefazolin was administered for antibiotic prophylaxis at a dose of 1 g for patients weighing <80 kg and 2 g for those weighing ≥80 kg. Postoperatively, cefazolin 1 g was administered every 8 h for 24 h and then discontinued. A pneumatic thigh tourniquet inflated to 300 mmHg was used in all cases.

Surgery began with diagnostic arthroscopy, during which the medial, lateral, and patellofemoral compartments were systematically evaluated and chondral and meniscal pathologies were documented. Concomitant intra-articular treatment was performed according to the arthroscopic findings. All identified medial meniscus posterior root tears (MMPRTs) were repaired. Other repairable medial meniscal tears were treated with meniscal repair, whereas nonrepairable medial meniscal pathology was treated with partial meniscectomy when indicated. In patients undergoing partial meniscectomy, concomitant chondroplasty and microfracture were also performed for associated medial-compartment chondral lesions when indicated. Thus, more than one intra-articular procedure could be performed in the same patient. Following arthroscopy, 1 g of tranexamic acid diluted in 100 mL of 0.9% sodium chloride was administered by slow intravenous infusion.

After diagnostic arthroscopy and completion of the correction-score assessment described in Section 2.3, coronal mechanical alignment was verified intraoperatively under fluoroscopic guidance using the electrocautery-cord cable technique [3,4]. The cable was aligned with the center of the ankle distally and the center of the femoral head proximally, and its passage across the tibial plateau was assessed with the knee in a true anteroposterior projection relative to the selected correction direction (Figure 3).

Before osteotomy, two Kirschner wires were inserted parallel to the proximal tibial joint surface as protective stop wires, and two additional Kirschner wires were placed under fluoroscopic guidance from the medial proximal tibia toward the fibular head to guide the osteotomy (Figure 4a). Through a medial approach, the superficial medial collateral ligament was carefully released, and a biplanar MOWHTO was performed beginning approximately 3 cm distal to the joint line. A lateral cortical bone bridge of approximately 10 mm was preserved to maintain lateral-hinge integrity, and the anterior osteotomy cut was completed with the tibial tubercle remaining attached to the distal fragment [3,4] (Figure 4b).

The osteotomy was gradually opened using a standard distractor (Figure 4c). Based on the Miniaci planning framework and selected correction direction, the available fixed wedge height closest to the required osteotomy opening was selected. In the present cohort, a TomoFix-type medial proximal tibial locking plate system with fixed wedge heights of 10 or 12.5 mm (Trumed Medical^®^, Türkiye) was used.

To minimize unintended changes in posterior tibial slope (PTS), the plate wedge was positioned at approximately the posterior three-quarters of the osteotomy gap in the sagittal plane, and fixation was completed with locking screws (Figure 4d,e). Posterior positioning of the fixation construct and preservation of an appropriate lateral hinge are recognized technical considerations for limiting unintended PTS changes during MOWHTO [20].

When grafting was indicated according to the selective strategy described in Section 2.3, an autologous tricortical graft harvested from the ipsilateral anterior iliac crest was inserted into the osteotomy gap. No graft material was used in cases managed without grafting. After fixation, implant position, osteotomy gap, lateral cortical-hinge integrity, and final mechanical alignment were reassessed fluoroscopically. A Hemovac drain was inserted, and the surgical wound was closed in anatomical layers.

### 2.5. Postoperative Rehabilitation

A standardized postoperative rehabilitation framework was used for all patients, irrespective of graft use, with procedure-specific modification of knee range of motion after meniscal repair. A hinged knee brace was used during mobilization for the first 4 weeks. In patients who underwent medial meniscus posterior root tear (MMPRT) repair or other medial meniscal repair, knee flexion was restricted to 90° for the first 45 days.

Quadriceps-strengthening exercises, ankle-pump exercises, and active and passive knee range-of-motion exercises within the permitted range were initiated on the first postoperative day. Progressive partial weight-bearing was permitted for the first 45 days, followed by advancement to full weight-bearing according to radiographic healing and clinical tolerance. The weight-bearing protocol did not differ according to concomitant intra-articular treatment or graft use.

For venous thromboembolism prophylaxis, low-molecular-weight heparin was administered during hospitalization, followed by acetylsalicylic acid (100 mg once daily) for 30 days after discharge. Bilateral thigh-high anti-embolism stockings were used for the first 2 postoperative weeks.

Clinical and radiographic follow-up examinations were performed at 2, 6, and 12 weeks; at 6 and 12 months; and annually thereafter.

### 2.6. Clinical Assessment

Clinical outcomes were assessed preoperatively and at the final follow-up using the validated Turkish-language versions of the International Knee Documentation Committee (IKDC) Subjective Knee Form [21] and the Lysholm Knee Score [22], together with the Visual Analog Scale (VAS). The IKDC and Lysholm scores are established patient-reported measures of knee function and have also been used for longitudinal assessment of functional outcomes following MOWHTO [23]. Pain intensity during activity was assessed using an 11-point VAS ranging from 0 to 10, with 0 indicating no pain and 10 indicating the worst imaginable pain. Changes in IKDC and Lysholm scores were calculated as the final follow-up value minus the preoperative value, whereas VAS improvement was calculated as the preoperative value minus the final follow-up value; thus, positive values indicated clinical improvement.

All clinical outcome assessments were performed face-to-face during routine outpatient follow-up visits by the same clinical team. Telephone or electronic assessments were not used, and the same mode of outcome assessment was applied throughout the study period. At the time the clinical scores were obtained, the clinical team performing the outcome assessments was blinded to postoperative radiographic measurements, including HKA, and to the selected preoperative correction direction.

The final follow-up IKDC score was designated as the primary clinical outcome for the present analysis. Final follow-up Lysholm and VAS scores, together with changes in IKDC, Lysholm, and VAS scores from baseline to final follow-up, were considered secondary clinical outcomes.

### 2.7. Radiological Assessment

Radiological assessments were performed using standing weight-bearing full-length lower-extremity radiographs and standard anteroposterior and lateral knee radiographs. Preoperative and postoperative radiographic measurements were performed digitally using the institutional picture archiving and communication system (PACS). Standing full-length lower-extremity radiographs were obtained according to a standardized institutional acquisition protocol with a source-to-image distance of 305 cm. Patients were positioned without a knee brace, with the feet approximately shoulder-width apart, both knees in full extension, and the patellae oriented directly forward to minimize rotational malpositioning. Radiographs were obtained without footwear, and patients were instructed to bear full weight on both lower extremities during image acquisition. Particular attention was paid to maintaining a standardized bilateral stance and avoiding protective unloading of the operated limb. Although full bilateral weight-bearing was instructed, the actual distribution of load between the two lower extremities was not quantitatively monitored.

The 6-week radiographic assessment represented the routine clinical follow-up window rather than a fixed postoperative day; therefore, the exact timing varied slightly among patients around the 6-week visit. Although progressive partial weight-bearing during routine ambulation was maintained for the first 45 postoperative days, patients were instructed to bear full weight bilaterally for the brief period required to obtain the standing full-length radiograph at this follow-up assessment, including those who were still completing the progressive weight-bearing phase of the postoperative rehabilitation protocol.

Coronal and sagittal alignment was evaluated using the hip–knee–ankle angle (HKA), medial proximal tibial angle (MPTA), lateral distal femoral angle (LDFA), and posterior tibial slope (PTS). HKA, MPTA, and LDFA were measured on full-length lower-extremity radiographs, whereas PTS was measured on lateral knee radiographs [15].

Postoperative 6-week HKA was defined as the primary radiological exposure variable and was expressed in degrees (°), with varus values designated as negative and valgus values as positive. Representative examples of postoperative mechanical alignment are shown in Figure 5. Analyses in which HKA was modeled as a continuous or categorical variable are described in the Statistical Analysis Section.

Maintenance of coronal mechanical correction over time was assessed by comparing HKA and MPTA values at 6 weeks postoperatively with those obtained at the final follow-up. Changes in HKA and MPTA were calculated as the final follow-up value minus the postoperative 6-week value, whereas the change in PTS was calculated as the postoperative value minus the preoperative value. Accordingly, a negative change in HKA indicated a reduction in valgus alignment during follow-up. For the maintenance-of-correction analysis, an absolute change of ≥3° in HKA or MPTA between the 6-week and final follow-up measurements was operationally defined as substantial loss of correction. Lateral cortical-hinge integrity was assessed using intraoperative fluoroscopy and early postoperative radiographs, and lateral-hinge fractures were recorded as perioperative events [18].

All available radiographic measurements were independently re-evaluated by two orthopedic surgeons who were blinded to the clinical outcomes. Observer-specific measurements were recorded separately and retained for formal interobserver reliability analysis. Measurements requiring reconciliation were subsequently jointly re-evaluated by the two assessors, and the consensus value was entered into the finalized radiographic dataset used for the main analyses; the two independent measurements were not automatically averaged. Interobserver reliability was formally assessed for HKA, MPTA, LDFA, and PTS using the independent measurements of both observers.

### 2.8. Osteotomy Union Assessment

Osteotomy union was assessed using serial anteroposterior and lateral knee radiographs together with clinical evaluation. Radiographic union was defined by trabecular bone bridging across the osteotomy site with progressive loss of visibility of the osteotomy line, whereas clinical union was defined as the ability to bear full weight without pain at the osteotomy site. Union was considered to have occurred when both radiographic and clinical criteria were fulfilled [24]. Representative progression of radiographic healing during serial follow-up is shown in Figure 6.

Time to union was recorded as the interval from surgery to the first scheduled follow-up assessment at which both radiographic and clinical union criteria were fulfilled. Because union could only be identified at discrete follow-up assessments, the exact biological time of union was not directly observed; therefore, the recorded value represents the first documented assessment at which union criteria were satisfied.

Delayed union was defined as failure to meet the union criteria by 6 months postoperatively despite evidence of progressive bone healing on serial radiographs, with subsequent achievement of union during further follow-up. Nonunion was defined as failure to meet the union criteria by 12 months postoperatively in the absence of progressive radiographic healing [24].

### 2.9. Assessment of Perioperative and Postoperative Complications

Perioperative and postoperative osteotomy-related complications were recorded throughout the follow-up period. Events of interest included lateral cortical-hinge fracture, prolonged wound drainage, microbiologically confirmed postoperative infection, delayed union, nonunion, and the need for revision surgery. Substantial loss of mechanical correction was assessed separately using the radiographic definition described in Section 2.7.

Prolonged wound drainage was defined as persistent drainage from the surgical wound for more than 3 postoperative days. In patients with drainage persisting beyond this period, wound cultures were obtained. Patients with prolonged wound drainage but negative cultures were managed with local wound care and were not classified as having a microbiologically confirmed infection. Culture-positive patients were initially managed with local wound care and systemic antibiotic therapy. Surgical debridement was performed when wound drainage persisted despite these measures. Implant retention was attempted in these cases, and no implant was removed for infection during the study period.

Donor-site morbidity after iliac crest graft harvest, including donor-site pain, hematoma, and harvest-site infection, was not systematically captured as a predefined variable in the retrospective dataset and therefore could not be reliably quantified.

Mechanical revision was defined as revision osteotomy or revision of fixation performed because of implant failure, loss of correction, or other osteotomy-related mechanical failure. Surgical debridement performed for infection control was evaluated separately and was not classified as mechanical revision. Complications were assessed on the basis of clinical examination and serial radiographs, with additional imaging obtained when clinically indicated.

### 2.10. Statistical Analysis

The distribution of continuous variables was assessed using the Shapiro–Wilk test, histograms, and Q–Q plots. Normally distributed continuous variables are presented as mean ± standard deviation (SD), non-normally distributed variables as median and interquartile range (IQR), and categorical variables as number and percentage [n (%)]. Between-group comparisons were performed using the independent-samples *t*-test or Mann–Whitney U test for continuous variables, as appropriate, and the chi-square test or Fisher’s exact test for categorical variables. Within-group comparisons of preoperative and final follow-up clinical scores were performed using the paired-samples *t*-test or Wilcoxon signed-rank test, according to data distribution.

No formal a priori sample-size calculation was performed because of the retrospective design. All patients meeting the predefined eligibility criteria during the study period and with the data required for the primary analyses were included, resulting in a final analytical cohort of 188 patients. The primary multivariable models contained 11 estimated predictor parameters, excluding the intercept, corresponding to approximately 17.1 observations per estimated parameter. This ratio was considered a descriptive assessment of model complexity rather than evidence that overfitting was absent. Accordingly, multivariable estimates were interpreted together with their 95% confidence intervals (CIs), model diagnostics, and sensitivity analyses.

The final follow-up IKDC score was designated as the primary clinical outcome for the present analysis. Final follow-up Lysholm and VAS scores, together with changes in IKDC, Lysholm, and VAS scores, were considered secondary clinical outcomes. Changes in IKDC and Lysholm scores were calculated as final follow-up value − preoperative value, whereas VAS improvement was calculated as preoperative value − final follow-up value, such that positive values represented clinical improvement.

The primary exposure variable was postoperative 6-week HKA. HKA was expressed in degrees (°), with negative values indicating varus and positive values indicating valgus alignment. In the primary analysis, postoperative HKA was modeled as a continuous variable, and its association with the final follow-up IKDC score was evaluated per 1° increase toward valgus. The same approach was applied secondarily to final follow-up Lysholm and VAS scores.

Multivariable linear regression models were constructed separately for final follow-up IKDC, Lysholm, and VAS scores. Postoperative 6-week HKA was the primary independent variable in all models. The primary models were adjusted for age, sex, body mass index (BMI), follow-up duration, preoperative HKA, Kellgren–Lawrence (KL) grade, medial-compartment International Cartilage Repair Society (ICRS) grade, medial meniscus posterior root tear (MMPRT), other medial meniscal pathology, and the corresponding preoperative clinical score. These variables were selected on the basis of their clinical relevance and their potential relationships with postoperative alignment and clinical outcomes. Operative variables arising within the treatment pathway, including osteotomy opening size, lateral cortical-hinge fracture, and graft use, were not included in the primary adjustment set because they could represent treatment-related, mediating, or collider variables rather than conventional baseline confounders. The original broader models including these operative variables were retained as sensitivity analyses to assess whether their inclusion materially altered the HKA–outcome estimates.

As a sensitivity analysis addressing potential confounding by the intended correction direction, the primary multivariable models were additionally adjusted for the algorithm-derived target-direction category (neutral/slight-valgus-oriented [correction score 0–1] vs. Fujisawa-oriented [correction score 2–4]). Because patient-specific numerical planned HKA values and weight-bearing-line percentages were not systematically recorded, this categorical variable represented the available measure of the intended correction direction.

To address potential confounding by concomitant intra-articular treatment, an additional sensitivity analysis was performed using a four-category treatment-pattern variable comprising diagnostic arthroscopy only, MMPRT repair, other medial meniscal repair, and partial meniscectomy with concomitant chondroplasty and microfracture. These treatment-pattern categories were mutually exclusive at the patient level, although more than one procedure could be performed within the partial meniscectomy/chondroplasty/microfracture treatment pattern. In this sensitivity analysis, the treatment-pattern variable replaced the MMPRT and other medial meniscal pathology variables, while the remaining primary-model covariates were retained.

The primary multivariable analyses were performed using a complete-case approach. Among the 188 patients included in the final analytical cohort, complete data were available for the primary exposure, clinical outcomes, and all covariates included in the principal regression models; therefore, no imputation of missing data was required.

Potential nonlinear associations between postoperative HKA and clinical outcomes were explored using supplementary regression models containing both linear and quadratic HKA terms (HKA and HKA^2^). A statistically significant quadratic term was considered evidence of departure from linearity. If a significant quadratic association was identified, the turning point of the fitted regression equation was calculated as −β_1_/(2β_2_) and reported only when it fell within the observed HKA range. These analyses were exploratory, and any estimated turning point was not interpreted as a causal optimum or clinical correction target.

As a supplementary descriptive analysis, postoperative 6-week HKA was also examined categorically. Based on the observed HKA distribution, patients were classified as near-neutral/slight valgus (HKA < 2.0°; n = 44), intermediate valgus (HKA 2.0–2.9°; n = 39), or greater valgus (HKA ≥ 3.0°; n = 105), with observed HKA ranges of 0.4–1.5°, 2.0–2.9°, and 3.0–4.5°, respectively. Final follow-up IKDC, Lysholm, and VAS scores and their corresponding change scores were compared across these categories using one-way analysis of variance (ANOVA) or the Kruskal–Wallis test, as appropriate. When a significant overall group difference was identified, pairwise post-hoc comparisons were performed using Holm-adjusted *p*-values to control the family-wise Type I error rate. These categorical analyses were considered supplementary and descriptive; principal inferences were based on the multivariable models treating postoperative HKA as a continuous variable.

Maintenance of mechanical correction was assessed by comparing HKA and MPTA at 6 weeks postoperatively and at final follow-up. Changes in HKA and MPTA were calculated as final follow-up value − postoperative 6-week value; accordingly, a negative HKA change indicated a reduction in valgus alignment during follow-up. Mean longitudinal changes in HKA and MPTA were reported with 95% CIs. At the individual-patient level, an absolute angular change of ≥3° between the 6-week postoperative and final follow-up measurements was operationally defined as substantial loss of correction. This pragmatic threshold was informed by the previous HTO literature in which an absolute change of ≥3° in MPTA during follow-up was used to define loss of correction [25]. The number and proportion of patients meeting this threshold were calculated separately for HKA and MPTA.

Time to union, maintenance of mechanical alignment, and perioperative/postoperative complications were considered secondary outcomes. Graft use was not treated as the primary exposure or as a grouping variable for the main analysis. Exploratory unadjusted comparisons according to graft use were performed for time to union and longitudinal changes in HKA and MPTA; these analyses were not intended to estimate a causal effect of grafting.

Interobserver reliability of the radiographic measurements was assessed using the independent measurements of the two observers for the entire cohort (n = 188). Intraclass correlation coefficients (ICCs) were calculated using a two-way random-effects model with an absolute-agreement definition. Ninety-five percent CIs for the ICC estimates were obtained using 10,000 bootstrap resamples. Reliability was assessed separately for HKA, MPTA, LDFA, and PTS at the available assessment time points. ICC values were interpreted as <0.50 indicating poor reliability, 0.50–0.75 moderate reliability, 0.75–0.90 good reliability, and >0.90 excellent reliability.

Assumptions of the linear regression models were assessed using residual plots and Q–Q plots. Heteroskedasticity was evaluated using the Breusch–Pagan test, multicollinearity using variance inflation factors (VIFs), and model specification using the Ramsey RESET test. Potentially influential observations were assessed using studentized residuals, leverage values, and Cook’s distance. HC3 heteroskedasticity-robust standard errors were used to provide inference that was more robust to potential heteroskedasticity [26]. Regression results are reported as unstandardized regression coefficients (β), robust standard errors (SEs), 95% CIs, and *p*-values. Sensitivity analyses excluding potentially influential observations identified by these diagnostics were additionally performed to assess the robustness of the primary findings.

For the primary outcome, a two-sided *p* < 0.05 was considered statistically significant. No global multiplicity adjustment was applied across the primary and secondary regression outcomes because final follow-up IKDC was designated as the single primary clinical outcome for the present analysis. Lysholm, VAS, change-score, nonlinear, categorical, union, and maintenance-of-correction analyses were considered secondary, exploratory, or supportive, as applicable. Accordingly, these analyses were interpreted with emphasis on effect estimates and 95% CIs rather than solely on nominal *p*-values. Holm-adjusted *p*-values were used for post-hoc pairwise comparisons following the categorical HKA analyses.

Statistical analyses were performed using IBM SPSS Statistics for Windows, Version 29.0 (IBM Corp., Armonk, NY, USA) and R (R Foundation for Statistical Computing, Vienna, Austria).

## 3. Results

### 3.1. Patient Characteristics and Postoperative Mechanical Alignment

After application of the eligibility criteria, 188 patients were included in the final analysis (Figure 1). The mean age was 52.64 ± 4.74 years, and the mean follow-up duration was 25.48 ± 6.80 months (range, 12.0–45.7 months). Complete data for the primary exposure, clinical outcomes, and all covariates included in the primary multivariable models were available for all 188 patients; therefore, the complete-case regression analyses included the entire final cohort, with no observations excluded because of missing model data.

Based on the predefined individualized correction-score algorithm, 19 patients (10.1%) had a score of 0, 66 (35.1%) had a score of 1, 80 (42.6%) had a score of 2, 22 (11.7%) had a score of 3, and 1 (0.5%) had a score of 4. Accordingly, 85 patients (45.2%) were classified as neutral/slight-valgus-oriented (score 0–1), whereas 103 patients (54.8%) were classified as Fujisawa-oriented (score 2–4).

Regarding concomitant intra-articular treatment, 119 patients (63.3%) underwent diagnostic arthroscopy only, 15 (8.0%) underwent MMPRT repair, 14 (7.4%) underwent other medial meniscal repair, and 40 (21.3%) underwent partial meniscectomy with concomitant chondroplasty and microfracture. These four treatment-pattern categories were mutually exclusive at the patient level; within the latter category, partial meniscectomy, chondroplasty, and microfracture were components of the same combined treatment pattern.

The mean HKA changed from −9.45 ± 2.06° preoperatively to 2.88 ± 1.21° of valgus at 6 weeks postoperatively and 2.38 ± 1.32° of valgus at final follow-up. Postoperative 6-week HKA values ranged from 0.4° to 4.5° of valgus. In the supplementary categorical analysis, 44 patients (23.4%) were classified as having near-neutral/slight valgus alignment (HKA < 2.0°), 39 (20.7%) as having intermediate valgus alignment (HKA 2.0–2.9°), and 105 (55.9%) as having greater valgus alignment (HKA ≥ 3.0°). The demographic, clinical, surgical, and radiological characteristics of the study cohort are summarized in Table 2.

Interobserver reliability was excellent for HKA, MPTA, and PTS measurements. For the primary radiographic exposure, 6-week postoperative HKA, the ICC was 0.999 (95% CI, 0.999–1.000). Preoperative and final-follow-up HKA measurements also demonstrated excellent reliability (ICC = 0.999 for both). MPTA measurements demonstrated excellent agreement across all assessment time points (ICC = 1.000), while PTS reliability was excellent preoperatively (ICC = 0.994) and postoperatively (ICC = 0.980). In contrast, LDFA demonstrated moderate reliability preoperatively (ICC = 0.696) and at 6 weeks postoperatively (ICC = 0.651), and poor reliability at final follow-up (ICC = 0.422). Detailed ICC estimates with 95% CIs are provided in Supplementary Table S1.

### 3.2. Clinical Outcomes

Significant improvements were observed across all clinical outcome measures following MOWHTO. The mean IKDC score increased from 39.37 ± 8.45 preoperatively to 80.11 ± 8.26 at the final follow-up, while the mean Lysholm score increased from 47.46 ± 8.60 to 79.26 ± 10.27. The mean VAS score decreased from 6.99 ± 0.95 to 1.82 ± 0.40 (all *p* < 0.001). The magnitude of improvement in each clinical outcome, together with the corresponding 95% confidence intervals (CIs), is presented in Table 3.

### 3.3. Association Between Postoperative HKA and Early- to Mid-Term Clinical Outcomes

In the multivariable analyses, each 1° increase in valgus HKA at 6 weeks postoperatively was independently associated with a 1.49-point-higher final follow-up IKDC score, the primary clinical outcome (β = 1.49; 95% CI, 0.04–2.93; *p* = 0.044). Similarly, each 1° increase in valgus HKA was associated with a 2.61-point-higher final follow-up Lysholm score (β = 2.61; 95% CI, 1.10–4.13; *p* < 0.001). In contrast, no statistically significant independent association was observed between postoperative HKA and final follow-up VAS score (β = 0.004; 95% CI, −0.050 to 0.058; *p* = 0.883) (Table 4).

In the crude analyses, each 1° increase in postoperative valgus HKA was associated with a 1.30-point-higher final IKDC score (95% CI, 0.16–2.44; *p* = 0.026) and a 3.69-point-higher final Lysholm score (95% CI, 2.53–4.86; *p* < 0.001), whereas no significant crude association was observed with final VAS (β = 0.008; 95% CI, −0.038 to 0.055; *p* = 0.723). After multivariable adjustment, the positive association remained statistically significant for IKDC, was attenuated but remained statistically significant for Lysholm, and remained non-significant for VAS. Complete coefficient estimates, HC3-robust 95% CIs, and *p*-values for all covariates included in the three primary multivariable models are provided in Supplementary Table S2.

Model diagnostics showed no evidence of problematic multicollinearity, with maximum variance inflation factor values below 2.34 across the primary outcome models. The Breusch–Pagan test indicated evidence of heteroskedasticity in the IKDC (*p* = 0.015), Lysholm (*p* = 0.016), and VAS (*p* = 0.039) models; accordingly, HC3 heteroskedasticity-robust standard errors were used for inference. The Ramsey RESET test did not indicate statistically significant functional-form misspecification for the IKDC (*p* = 0.462), Lysholm (*p* = 0.913), or VAS (*p* = 0.118) models. Maximum leverage values were 0.138, 0.150, and 0.148, and maximum Cook’s distances were 0.081, 0.084, and 0.095 for the IKDC, Lysholm, and VAS models, respectively. No observation had a Cook’s distance >1, indicating that no single observation exerted extreme influence on the fitted models.

In the outcome-specific residual sensitivity analyses, one observation in the IKDC model, two in the Lysholm model, and four in the VAS model had absolute externally studentized residuals > 3. After exclusion of these observations and refitting of the same multivariable models, the HKA coefficient remained positive but was attenuated in the IKDC model (β = 1.19; 95% CI, −0.18 to 2.55; *p* = 0.088), whereas the association remained statistically significant for Lysholm (β = 2.51; 95% CI, 1.15–3.87; *p* < 0.001). The HKA–VAS association remained non-significant (β = 0.003; 95% CI, −0.047 to 0.054; *p* = 0.900). Thus, the direction of the HKA coefficient was preserved across these sensitivity analyses, although the statistical evidence for the primary IKDC association was sensitive to exclusion of the observation with a large residual.

In the sensitivity analysis additionally adjusting for the algorithm-derived target-direction category (neutral/slight-valgus-oriented, n = 85; Fujisawa-oriented, n = 103), the association between postoperative HKA and clinical outcomes remained materially unchanged. Each 1° increase in valgus HKA remained associated with a 1.57-point-higher final IKDC score (95% CI, 0.12–3.02; *p* = 0.033) and a 2.46-point-higher final Lysholm score (95% CI, 0.90–4.03; *p* = 0.002), whereas no statistically significant association was observed for final VAS (β = 0.007; 95% CI, −0.047 to 0.062; *p* = 0.798). The target-direction category itself was not independently associated with final IKDC (β = 1.70; *p* = 0.367), final Lysholm (β = −2.95; *p* = 0.166), or final VAS (β = 0.061; *p* = 0.498).

In the additional sensitivity analysis accounting for concomitant intra-articular treatment, the association between postoperative HKA and clinical outcomes remained materially unchanged. After replacing the MMPRT and other medial meniscal pathology variables with the four-category intra-articular treatment-pattern variable, each 1° increase in valgus HKA remained associated with a 1.49-point-higher final IKDC score (95% CI, 0.05–2.94; *p* = 0.043) and a 2.61-point-higher final Lysholm score (95% CI, 1.10–4.13; *p* < 0.001). No statistically significant association was observed with final VAS (β = 0.004; 95% CI, −0.050 to 0.058; *p* = 0.882). These findings indicated that accounting for concomitant intra-articular treatment did not materially alter the observed HKA–outcome associations.

In the supplementary nonlinear analyses, the quadratic HKA term (HKA^2^) was not statistically significant in the models for IKDC (*p* = 0.245), Lysholm (*p* = 0.303), or VAS (*p* = 0.914). Accordingly, within the observed range of 0.4–4.5° of valgus, no statistically detectable nonlinearity was identified for any of the evaluated clinical outcomes.

### 3.4. Clinical Outcomes According to Postoperative Mechanical Alignment Categories

In the supplementary descriptive categorical analyses, no significant differences were observed among the postoperative HKA categories in either final follow-up IKDC score or change in IKDC (ΔIKDC) (*p* = 0.125 and *p* = 0.152, respectively). In contrast, both final follow-up Lysholm score and change in Lysholm (ΔLysholm) differed significantly across the alignment categories (both *p* < 0.001).

In Holm-adjusted pairwise comparisons, final follow-up Lysholm score was significantly higher in both the intermediate-valgus and greater-valgus groups than in the near-neutral/slight-valgus group (both adjusted *p* < 0.001), whereas no significant difference was observed between the intermediate- and greater-valgus groups (adjusted *p* = 0.852). For ΔLysholm, only the difference between the near-neutral/slight-valgus and greater-valgus groups remained statistically significant after Holm adjustment (adjusted *p* < 0.001).

Neither final follow-up VAS score nor VAS improvement differed significantly among the HKA categories (*p* = 0.450 and *p* = 0.464, respectively). These categorical analyses were supplementary and descriptive and were not intended to identify independent clinical thresholds or an optimal postoperative HKA. Detailed group values and comparisons are presented in Table 5.

### 3.5. Maintenance of Mechanical Correction During Follow-Up and Radiological Outcomes

Small longitudinal changes in coronal alignment were observed during follow-up. From 6 weeks postoperatively to final follow-up, the mean change in HKA was −0.50 ± 0.28° (95% CI, −0.54 to −0.46°; *p* < 0.001), while the mean change in MPTA was −0.39 ± 0.23° (95% CI, −0.42 to −0.36°; *p* < 0.001). At the individual-patient level, no patient demonstrated an absolute change of ≥3° in either HKA or MPTA between the 6-week postoperative and final follow-up assessments (0/188, 0% for both measures). The surgery-related change in PTS was 0.20 ± 0.80° (95% CI, 0.09–0.31°; *p* = 0.001). Although these mean angular changes were statistically significant, their absolute magnitudes were small and should not be interpreted as clinically substantial changes solely on the basis of statistical significance (Table 6).

Union was achieved in all patients, with no cases of delayed union or nonunion. Because union was identified at scheduled follow-up assessments rather than by continuous observation, the exact biological time of union could not be determined. The median time to the first follow-up assessment documenting fulfillment of the predefined union criteria was 12.2 weeks (range, 10.0–17.8 weeks) (Table 6).

Autologous tricortical iliac crest grafting was used in 96 of 188 patients (51.1%). According to osteotomy opening size, grafting was performed in 50 of 118 patients (42.4%) with a 10-mm opening and in 46 of 70 patients (65.7%) with a 12.5-mm opening. Among the 24 patients with a lateral cortical hinge fracture, grafting was used in 16 patients (66.7%), compared with 80 of 164 patients (48.8%) without a documented hinge fracture. By hinge-fracture type, grafting was used in 12 of 17 type 1 fractures, 3 of 5 type 2 fractures, and 1 of 2 type 3 fractures. These distributions illustrate that graft use was not restricted to hinge-fracture cases and was selectively applied according to the combined intraoperative consideration of osteotomy opening size and lateral-hinge integrity.

In secondary unadjusted analyses according to graft use, no significant between-group differences were observed in time to first documented union, ΔHKA, or ΔMPTA (all *p* > 0.05). These analyses were exploratory and unadjusted and should not be interpreted as estimates of the causal effect of grafting. Maintenance of mechanical correction and other radiological outcomes are summarized in Table 6 and Table 7.

Lateral cortical hinge fracture occurred in 24 patients (12.8%), including 17 type 1, five type 2, and two type 3 fractures. Prolonged wound drainage (>3 postoperative days) occurred in 14 patients (7.4%), and wound cultures were obtained in all 14 cases. Ten patients (5.3%) had negative cultures and were successfully managed with local wound care alone; these cases were not classified as microbiologically confirmed postoperative infections.

The remaining four patients (2.1%) had culture-positive postoperative infections, with methicillin-resistant *Staphylococcus aureus* (MRSA) isolated in all four cases. These patients were initially managed with local wound care and systemic antibiotic therapy; however, because wound drainage persisted, all four subsequently underwent surgical debridement with implant retention. All four patients received a 2-week course of vancomycin and achieved resolution of infection. No implant removal was required because of infection, and union was ultimately achieved in all four patients.

No cases of delayed union, nonunion, or mechanical revision were observed during follow-up. Donor-site morbidity after iliac crest graft harvest was not systematically captured in the retrospective dataset; therefore, reliable rates of donor-site pain, hematoma, or harvest-site infection could not be reported. The detailed distribution of perioperative and postoperative complications is presented in Table 8.

## 4. Discussion

The principal finding of this study was that postoperative coronal mechanical alignment achieved after MOWHTO was associated with early- to mid-term functional outcomes. Within the observed postoperative HKA range of 0.4–4.5° of valgus, each 1° increase in valgus HKA was associated with a 1.49-point-higher final IKDC score and a 2.61-point-higher final Lysholm score after multivariable adjustment, whereas no significant association was observed with VAS. Supplementary categorical analyses provided supportive evidence primarily for the Lysholm outcome, while no significant between-group differences were observed for IKDC or VAS. In addition, the exploratory quadratic analyses showed no statistically detectable nonlinearity within the observed HKA range. Taken together, these findings demonstrate an observational association between achieved postoperative mechanical alignment and early- to mid-term functional outcomes within the restricted valgus range represented in this cohort, with a more pronounced and consistent association for Lysholm than for IKDC. These findings should not be interpreted as establishing a universal optimal HKA target or a causal benefit of increasing postoperative valgus.

### 4.1. Postoperative Mechanical Alignment and Clinical Outcomes

The fundamental biomechanical rationale of MOWHTO is to shift the weight-bearing axis away from the degenerative medial compartment, thereby reducing medial compartment loading [3,4]. The traditional Fujisawa concept advocates directing the weight-bearing line toward approximately 62.5% of the tibial plateau width [5], whereas more recent studies have suggested that the same degree of valgus correction may not be necessary for every patient and that near-neutral or slight-valgus alignment may provide satisfactory outcomes in appropriately selected patients [4,6,7,8]. Both insufficient correction and excessive valgus correction have also been associated with unfavorable outcomes [4,7,8].

The relationship between angular HKA correction and weight-bearing-line (WBL) position further illustrates the difficulty of defining a single universal valgus target. In a computer-based OWHTO simulation, correction through the conventional Fujisawa point corresponded to a median postoperative valgus angle of 2.4°, whereas a 4.5° valgus target shifted the mechanical axis further laterally to approximately 71.9% of the tibial plateau width [27]. Similarly, Kobayashi et al. reported a post-planning HKA of 2.9 ± 0.6° valgus when the WBL was targeted at 62.5% [5]. These findings indicate that HKA and WBL position are related but not directly interchangeable, and that their relationship may vary according to individual lower-limb anatomy. Accordingly, the upper limit of 4.5° valgus observed in our cohort should not be interpreted as an optimal target. The present study instead evaluated the association between the postoperative HKA actually achieved and subsequent clinical outcomes.

In the multivariable analyses, each 1° increase in postoperative valgus HKA was associated with a 1.49-point-higher final IKDC score and a 2.61-point-higher final Lysholm score. Previous studies have similarly demonstrated associations between postoperative coronal alignment and patient-reported or functional outcomes after HTO [11,12]. Blackburn et al. reported that coronal-plane alignment was related to patient-reported outcomes following HTO, although substantial heterogeneity in alignment definitions and outcome measures limited identification of a universally optimal target [12]. Changes across multiple lower-limb coronal alignment parameters after HTO have also been documented [13]. Our findings extend this evidence by evaluating achieved postoperative HKA as a continuous exposure while accounting for baseline clinical status and relevant pre-exposure characteristics.

The observed HKA associations remained materially unchanged in sensitivity analyses additionally accounting for the algorithm-derived intended correction direction and for concomitant intra-articular treatment. Nevertheless, the association with the primary IKDC outcome should be interpreted cautiously. The adjusted IKDC coefficient was 1.49 points per 1° increase in valgus HKA (95% CI, 0.04–2.93; *p* = 0.044), with the lower confidence bound close to the null. Across the observed HKA range of 0.4–4.5°, this coefficient corresponds to an estimated model-based difference of approximately 6.1 IKDC points. For context, an HTO-specific study reported an IKDC minimal clinically important difference (MCID) of approximately 12.5 points [28]. Because MCID primarily describes meaningful within-patient change and is not directly equivalent to an exposure-associated model estimate, this comparison is contextual rather than a formal test of clinical significance. Nevertheless, the magnitude of the IKDC association appears modest relative to this benchmark and should not be interpreted as demonstrating a large clinically important benefit for each additional degree of valgus.

The association was larger and more consistent for Lysholm. The supplementary categorical analyses showed significantly higher Lysholm outcomes in the intermediate- and greater-valgus groups than in the near-neutral/slight-valgus group, whereas final IKDC and ΔIKDC did not differ significantly across categories. This difference may partly reflect the distinct dimensions of knee function captured by these instruments [21,22,23] and the loss of information inherent in categorizing a continuous exposure. Accordingly, the primary interpretation should be based on the continuous multivariable HKA models, with the categorical analyses regarded as supplementary descriptive findings rather than evidence of independent clinical thresholds.

In contrast, postoperative HKA was not significantly associated with final VAS. Pain after MOWHTO is likely influenced by factors beyond coronal alignment, including intra-articular pathology, patellofemoral involvement, periarticular factors, and individual pain processing. Previous evidence has highlighted the heterogeneous and multifactorial nature of pain in knee osteoarthritis [29,30]. Final VAS scores in our cohort were also clustered near the lower end of the scale, potentially limiting discrimination according to postoperative alignment. Because pain sensitization, quantitative sensory testing, and myofascial abnormalities were not assessed in this cohort, however, these mechanisms cannot be inferred as explanations for the absence of an HKA–VAS association.

Importantly, our results do not establish an ideal postoperative HKA. The quadratic HKA term was not statistically significant for IKDC, Lysholm, or VAS; therefore, no statistically detectable nonlinearity was identified within the observed range. Moreover, the absence of a significant difference in final Lysholm scores between the intermediate- and greater-valgus categories argues against interpreting the results as evidence that progressively increasing valgus necessarily produces progressively better outcomes. This interpretation is consistent with the previous literature questioning the application of a single correction target to all patients [6,7,8,12]. Because postoperative HKA ranged only from 0.4° to 4.5° of valgus, these findings should not be extrapolated to more pronounced valgus alignment.

The findings are compatible with the concept that postoperative alignment may reasonably be considered in the context of patient- and knee-specific characteristics [4,6,7,8,10]. In our cohort, the intended correction direction was individualized using age, osteoarthritis severity, medial-compartment chondral damage, and medial meniscal pathology, with neutral/slight valgus and the Fujisawa point serving as reference directions. Thus, near-neutral alignment in some patients represented the intended correction direction rather than necessarily indicating technical undercorrection. However, patient-specific numerical target HKA or WBL values were not systematically recorded, and the study did not directly compare individualized planning with fixed neutral or fixed Fujisawa targeting. The present findings therefore cannot establish the superiority of individualized alignment planning.

Finally, postoperative HKA was not a randomized exposure but the result of preoperative deformity, correction planning, intra-articular pathology, patient characteristics, and surgical execution. Although the primary models adjusted for baseline clinical status and relevant pre-exposure covariates, confounding by indication and residual confounding cannot be excluded. The observed associations should therefore not be interpreted as a causal valgus dose–response relationship, but rather as adjusted observational associations between achieved postoperative HKA and early- to mid-term functional outcomes within the restricted valgus range represented in this cohort.

### 4.2. Maintenance of Mechanical Correction During Follow-Up

The coronal mechanical correction achieved in the early postoperative period was largely maintained during follow-up. Although the mean changes in HKA (−0.50°; 95% CI, −0.54 to −0.46°) and MPTA (−0.39°; 95% CI, −0.42 to −0.36°) were statistically significant, their absolute magnitudes were small. Moreover, no patient demonstrated an absolute change of ≥3° in either HKA or MPTA between the 6-week postoperative and final follow-up assessments. This threshold was used as a pragmatic indicator of substantial angular change, informed by the previous HTO literature in which an absolute change of ≥3° in MPTA during follow-up was used to define loss of correction [25]. Long-term MOWHTO data have also emphasized the potential clinical relevance of maintaining postoperative mechanical-axis alignment over time [2]. Although interobserver reliability was excellent for HKA and MPTA, high reader agreement does not eliminate potential variability related to standing radiographic acquisition, limb rotation, positioning, or weight distribution. Thus, the small statistically significant mean changes should not, by themselves, be interpreted as proof of mechanical stability.

The surgery-related change in PTS was also small (0.20°; 95% CI, 0.09–0.31°), despite reaching statistical significance. Because PTS was assessed preoperatively and postoperatively rather than longitudinally from 6 weeks to final follow-up, this finding reflects only a small surgery-related change in sagittal alignment rather than evidence of longitudinal sagittal stability. Previous biomechanical evidence has shown that osteotomy geometry, lateral-hinge orientation, and posterior plate positioning may influence preservation of PTS during medial opening-wedge osteotomy [20]. Ruzbarsky et al. demonstrated that a sagittally oriented lateral hinge combined with posterior plate placement and fixation with the knee in hyperextension could help maintain PTS across different magnitudes of coronal correction [20]. Although the surgical technique used in the present cohort incorporated principles intended to minimize unintended sagittal-plane changes, the study was not designed to isolate the effects of individual technical components. Therefore, the observed changes in coronal or sagittal alignment cannot be attributed to a specific technical factor or interpreted as evidence of superiority of the implant or surgical technique used in this study.

### 4.3. Union, Selective Bone Grafting, and Complications

Union was achieved in all patients, with no cases of delayed union or nonunion. This finding indicates a high rate of successful osteotomy healing within the 10–12.5-mm opening range examined in this cohort. Stable fixation and preservation of lateral cortical hinge integrity are recognized as important contributors to osteotomy stability and healing after MOWHTO [18,24,31], whereas the necessity of routinely filling the osteotomy gap with autograft, allograft, or bone substitutes remains controversial [19,32,33]. Nevertheless, the absence of delayed union or nonunion should not be interpreted as indicating a zero risk of impaired healing or superiority of the present approach over alternative techniques.

Autologous tricortical iliac crest grafting was used selectively rather than routinely, based primarily on osteotomy opening size and lateral-hinge integrity. In secondary unadjusted analyses, no significant differences were observed between grafted and nongrafted patients in time to first documented union, ΔHKA, or ΔMPTA. Previous comparative studies and evidence syntheses have shown heterogeneous findings regarding gap-filling strategies after MOWHTO [19,32,33]. In particular, although Mao et al. found no clear superiority of allograft over alternative strategies, some assessments suggested more favorable bone-healing progression with autograft [19]. Our findings should therefore not be interpreted as demonstrating equivalence between grafted and nongrafted osteotomies or as evidence that grafting is unnecessary. Because graft use was determined according to intraoperative considerations rather than randomized allocation, these comparisons are susceptible to confounding by indication. Furthermore, donor-site morbidity after iliac crest graft harvest was not systematically captured, precluding a complete assessment of the potential benefits and harms of autografting.

Lateral cortical hinge fracture occurred in 24 patients (12.8%), including 17 type 1, five type 2, and two type 3 fractures. Lateral hinge fracture is a recognized complication of MOWHTO and may compromise construct stability [18,31]. A systematic review demonstrated that computed tomography detects substantially more hinge fractures than conventional radiography, suggesting that studies relying predominantly on radiographic assessment may underestimate their true incidence [31]. The observed incidence should therefore be interpreted in the context of the imaging methods used for fracture detection.

Prolonged wound drainage occurred in 14 patients (7.4%); 10 were culture-negative and resolved with local wound care and were therefore not classified as microbiologically confirmed infections. Culture-positive postoperative infection occurred in four patients (2.1%), with MRSA isolated in all four cases. All four ultimately underwent surgical debridement with implant retention and systemic antibiotic therapy, achieved infection resolution and union, and required no infection-related implant removal. No mechanical revisions were observed. However, the small number of major complications precludes reliable assessment of associations between postoperative HKA and uncommon adverse events; therefore, these data do not establish whether particular postoperative HKA levels influence complication risk.

### 4.4. Clinical Implications

The findings of this study suggest that, after MOWHTO, not only correction of the preoperative varus deformity but also the postoperative mechanical alignment actually achieved was associated with early- to mid-term functional outcomes within the restricted alignment range represented in this cohort. Within the observed valgus range, a higher postoperative HKA was associated with better functional outcomes, particularly as assessed by the Lysholm score, whereas no corresponding association was demonstrated for pain. Thus, the clinical relevance of coronal alignment may differ according to the patient-reported outcome being considered.

However, the present findings should not be interpreted as supporting correction of all patients toward the Fujisawa point or toward progressively greater valgus. The necessity of the conventional Fujisawa target for every patient remains debated. In a prospective randomized study, Lin et al. reported comparable clinical outcomes between a more neutral target and the conventional Fujisawa-oriented target in patients undergoing HTO with medial meniscus posterior root tear [34]. More recent work has also explored individualized alignment targets based on patient-specific coronal alignment characteristics rather than applying a uniform correction target to all patients [10].

Importantly, the exploratory quadratic analyses showed no statistically detectable nonlinearity within the observed HKA range, which was restricted to 0.4–4.5° of valgus. These analyses therefore do not identify a specific optimal postoperative HKA value. Accordingly, the present results should not be interpreted according to a “more valgus is better” paradigm or extrapolated beyond the alignment range represented in this cohort. Instead, the findings support further investigation of individualized correction targets within the spectrum between neutral mechanical alignment and the Fujisawa point. Consistent with this concept, Erdem et al. demonstrated that postoperative mechanical-axis position and coronal-plane alignment phenotypes were associated with clinical outcomes after MOWHTO, further emphasizing that postoperative alignment should be interpreted in the context of individual coronal-plane characteristics [35].

Because of the observational design of the present study, these findings cannot establish the superiority of a particular HKA target or demonstrate that individualized alignment planning provides better outcomes than fixed-target strategies. Prospective studies directly comparing different correction targets across broader postoperative alignment ranges are required to determine which alignment ranges are most appropriate for specific patient subgroups.

### 4.5. Strengths

This study has several strengths. First, a relatively homogeneous cohort of 188 patients undergoing MOWHTO was evaluated. To reduce surgical heterogeneity, the analysis was restricted to patients treated using the same TomoFix-type locking plate system and osteotomy openings of 10 or 12.5 mm. All procedures were performed by a six-surgeon knee surgery team, including three primary surgeons, according to a common surgical protocol, with standardized postoperative rehabilitation.

Second, the primary exposure was the postoperative HKA actually achieved rather than the theoretically planned correction target. Treating HKA as a continuous variable in the primary analyses minimized the loss of information associated with categorization, while supplementary categorical and exploratory nonlinear analyses provided complementary assessments of the observed associations. Maintenance of correction was evaluated both by mean longitudinal changes in HKA and MPTA and at the individual-patient level using a pragmatic absolute angular-change threshold of ≥3°.

Third, the associations between postoperative HKA and clinical outcomes were evaluated using multivariable models adjusted for baseline clinical status and relevant pre-exposure covariates. Inclusion of the corresponding preoperative clinical score in each outcome model accounted for differences in baseline outcome status, while HC3 heteroskedasticity-robust standard errors provided inference robust to potential heteroskedasticity. Sensitivity analyses additionally accounted for the algorithm-derived intended correction direction and the pattern of concomitant intra-articular treatment, allowing for assessment of the robustness of the principal HKA–outcome associations.

Finally, radiographic measurements were independently evaluated by two orthopedic surgeons blinded to the clinical outcomes, and formal interobserver reliability analysis was performed. The primary radiographic exposure, 6-week postoperative HKA, demonstrated excellent interobserver reliability (ICC = 0.999; 95% CI, 0.999–1.000). HKA measurements at the other assessment time points, as well as MPTA and PTS measurements, also demonstrated excellent reliability. These findings reduce concern regarding reader-related variability in the primary radiographic exposure and support the reproducibility of the principal radiographic measurements used in the analyses.

### 4.6. Limitations

This study has several limitations. First, its retrospective, single-center design limits causal inference and generalizability. Exclusion of patients because of incomplete postoperative radiographs (n = 3), unavailable clinical scores (n = 16), incomplete preoperative data (n = 12), or follow-up of <12 months (n = 10) may also have introduced selection bias. Because comparable clinical and radiographic data were not available for all excluded patients, a formal comparison between included and excluded potentially eligible patients could not be performed. Postoperative HKA was not a randomized exposure but reflected preoperative deformity, correction planning, patient and intra-articular characteristics, and surgical execution. Although the primary models were adjusted for baseline clinical status and relevant pre-exposure covariates, confounding by indication and residual confounding cannot be excluded. Concomitant intra-articular treatment was addressed in a sensitivity analysis, but treatment allocation depended on the underlying pathology rather than randomization. In addition, individual rehabilitation adherence and physiotherapy exposure were not systematically quantified, and patients undergoing MMPRT or other medial meniscal repair had procedure-specific flexion restrictions. Several potentially relevant variables, including smoking status, diabetes mellitus, metabolic bone disease, baseline activity level, symptom duration, detailed preoperative range of motion, and the severity of lesser degrees of patellofemoral involvement, were also unavailable or incompletely characterized. Accordingly, the adjusted HKA associations should not be interpreted as causal effects.

Second, the primary exposure was HKA measured at 6 weeks postoperatively and therefore did not directly represent immediate postoperative alignment. HKA measured on standing full-length radiographs is also a static measure and does not capture dynamic loading during functional activities. Although only small longitudinal changes in HKA and MPTA were observed, these differences may partly reflect variability in standing radiographic acquisition, limb rotation, positioning, and weight distribution. Interobserver reliability was excellent for HKA, MPTA, and PTS, but LDFA demonstrated only moderate reliability preoperatively and at 6 weeks and poor reliability at final follow-up; findings involving LDFA should therefore be interpreted cautiously. Furthermore, patient-specific final planned HKA values and weight-bearing-line percentages were not systematically recorded. Consequently, planned-versus-achieved alignment and patient-level target accuracy could not be reliably quantified.

Third, postoperative HKA was restricted to 0.4–4.5° of valgus, with neither residual varus nor more-pronounced valgus represented. The observed associations therefore should not be extrapolated beyond this range or used to derive a universal optimal HKA target. Similarly, the <2.0°, 2.0–2.9°, and ≥3.0° categories used in supplementary analyses were descriptive, cohort-based categories rather than externally validated prognostic thresholds. The principal interpretation should therefore remain based on the continuous HKA analyses.

Fourth, although correction direction was individualized according to patient- and knee-specific characteristics, there was no independent comparator group treated using a fixed neutral, fixed Fujisawa, or alternative targeting strategy. The study therefore cannot establish the superiority of individualized alignment planning. This limitation is relevant given evidence of comparable outcomes with more neutral and conventional Fujisawa-oriented strategies and growing interest in patient-specific alignment targets [10,34,35].

Fifth, autologous tricortical iliac crest grafting was selectively applied rather than randomized, primarily according to osteotomy opening size and lateral-hinge integrity. Consequently, the secondary unadjusted comparisons between grafted and nongrafted patients are susceptible to confounding by indication and cannot establish the independent effect of grafting on time to first documented union or maintenance of correction. This caution is consistent with the heterogeneous evidence regarding gap-filling strategies after MOWHTO [19,32,33]. Donor-site morbidity was not systematically captured, precluding reliable assessment of donor-site pain, hematoma, harvest-site infection, or other graft-harvest complications. Postoperative wound complications were also identified retrospectively, which may have limited ascertainment of minor or incompletely documented events.

Sixth, to reduce surgical heterogeneity, the analysis was restricted to patients treated with the same TomoFix-type locking plate system and 10- or 12.5-mm osteotomy openings; the smaller numbers of patients with 8.5- or 15-mm openings were excluded. Although this increased cohort homogeneity, it limits generalizability to other opening magnitudes, fixation systems, surgical techniques, and rehabilitation protocols. Procedures were performed by a six-surgeon team, including three primary surgeons, using a common protocol; nevertheless, residual variability related to surgeon-specific technique, learning effects, or calendar time cannot be excluded.

Seventh, although the sample size was 188 patients and the primary models included 11 predictor parameters, providing approximately 17.1 observations per parameter, model instability cannot be completely excluded. The primary IKDC association was also sensitive to exclusion of the single observation with a large externally studentized residual. After exclusion, the HKA coefficient remained positive and of similar magnitude, but its 95% CI included zero (β = 1.19; 95% CI, −0.18 to 2.55; *p* = 0.088). This finding supports cautious interpretation of the IKDC association based on its effect estimate and uncertainty rather than nominal statistical significance alone. No global multiplicity adjustment was applied across secondary and supportive analyses; these findings should therefore be considered exploratory. Although HC3 heteroskedasticity-robust standard errors improve inference in the presence of heteroskedasticity, they do not eliminate potential model misspecification or instability.

Finally, the mean follow-up of approximately 2 years permitted assessment of early- to mid-term outcomes but was insufficient to determine long-term osteoarthritis progression, durability of correction, or conversion to total knee arthroplasty. Final clinical outcomes were assessed at variable follow-up intervals rather than at a uniform postoperative time point. Although follow-up duration was included in all primary multivariable models, residual confounding related to follow-up duration cannot be excluded. Long-term MOWHTO studies demonstrate that clinically relevant outcomes and survivorship extend well beyond the period examined here [2,23]. Patient-centered outcomes such as return to work or sport, activity level, satisfaction, and health-related quality of life were also not systematically assessed. Longer-term, prospective, and preferably multicenter studies incorporating broader postoperative alignment ranges and direct comparisons of correction strategies are needed to confirm these findings and identify appropriate alignment ranges for specific patient subgroups.

## 5. Conclusions

In this single-center retrospective cohort study, postoperative coronal mechanical alignment measured at 6 weeks after MOWHTO was associated with early- to mid-term patient-reported functional outcomes within the observed HKA range of 0.4–4.5° of valgus. Each 1° increase in valgus HKA was associated with a 2.61-point-higher final Lysholm score and a more modest 1.49-point-higher final IKDC score, whereas no statistically significant association was observed with final VAS.

The association was more pronounced and consistent for Lysholm, whereas the association with the primary outcome, IKDC, was modest and should be interpreted cautiously. Although the continuous multivariable association with IKDC reached statistical significance, the estimated model-based difference across the entire observed HKA range was approximately 6.1 points, and the supplementary categorical analysis did not demonstrate a significant between-group difference in IKDC. Exploratory quadratic analyses showed no statistically detectable nonlinearity within the observed HKA range.

Accordingly, these findings represent observational associations rather than a causal valgus dose–response relationship and do not establish a universal optimal postoperative HKA or the superiority of individualized correction over alternative targeting strategies. Postoperative HKA should therefore be regarded as a radiographic parameter associated with early- to mid-term functional outcomes within the restricted alignment range represented in this cohort. Prospective studies incorporating broader postoperative alignment ranges and direct comparisons of correction strategies are needed to determine the clinical relevance of specific alignment targets and to identify appropriate target ranges for different patient subgroups.

## Figures and Tables

**Figure 1:**
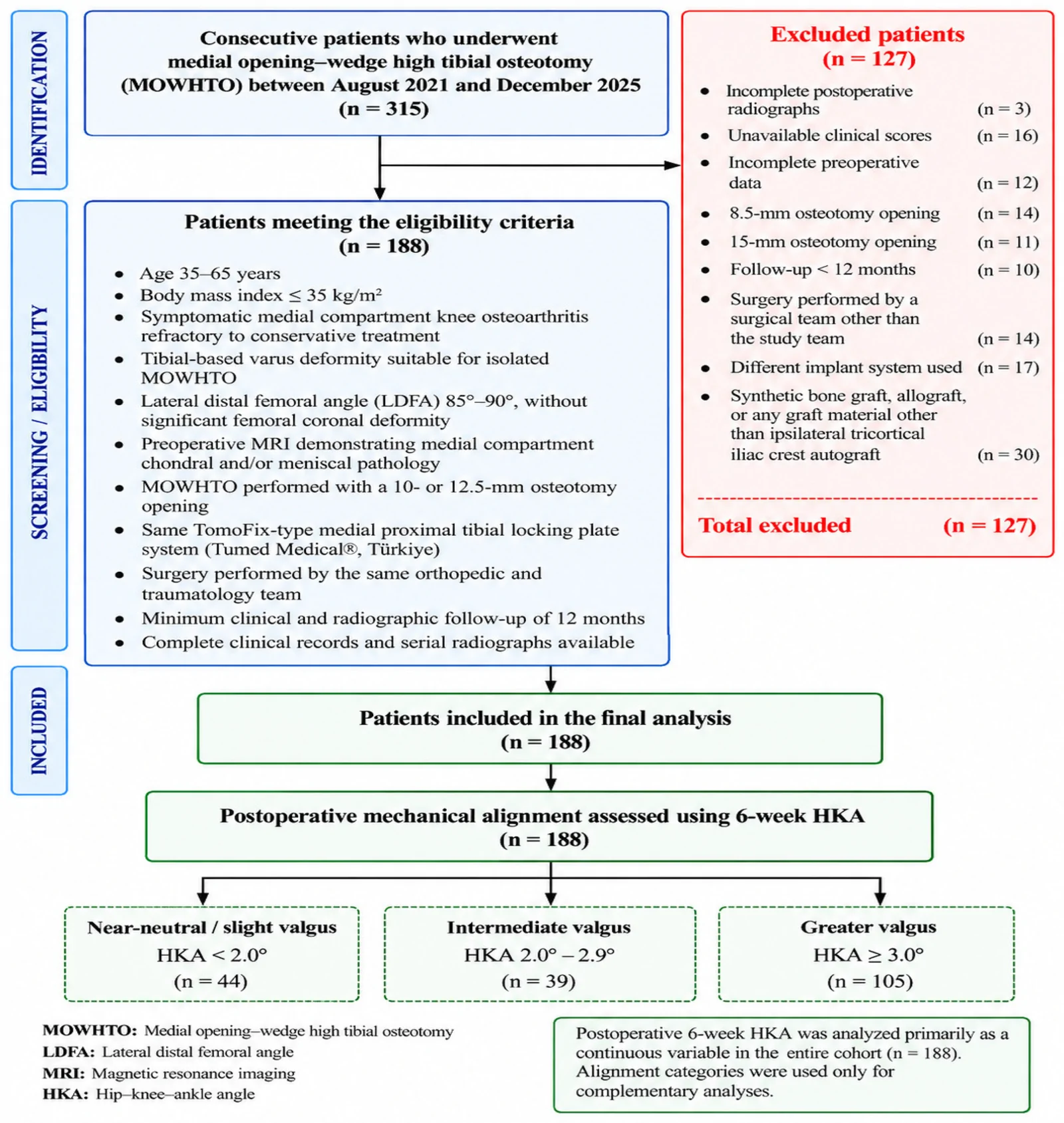
STROBE flow diagram illustrating patient selection and the formation of the final study cohort.

**Figure 2:**
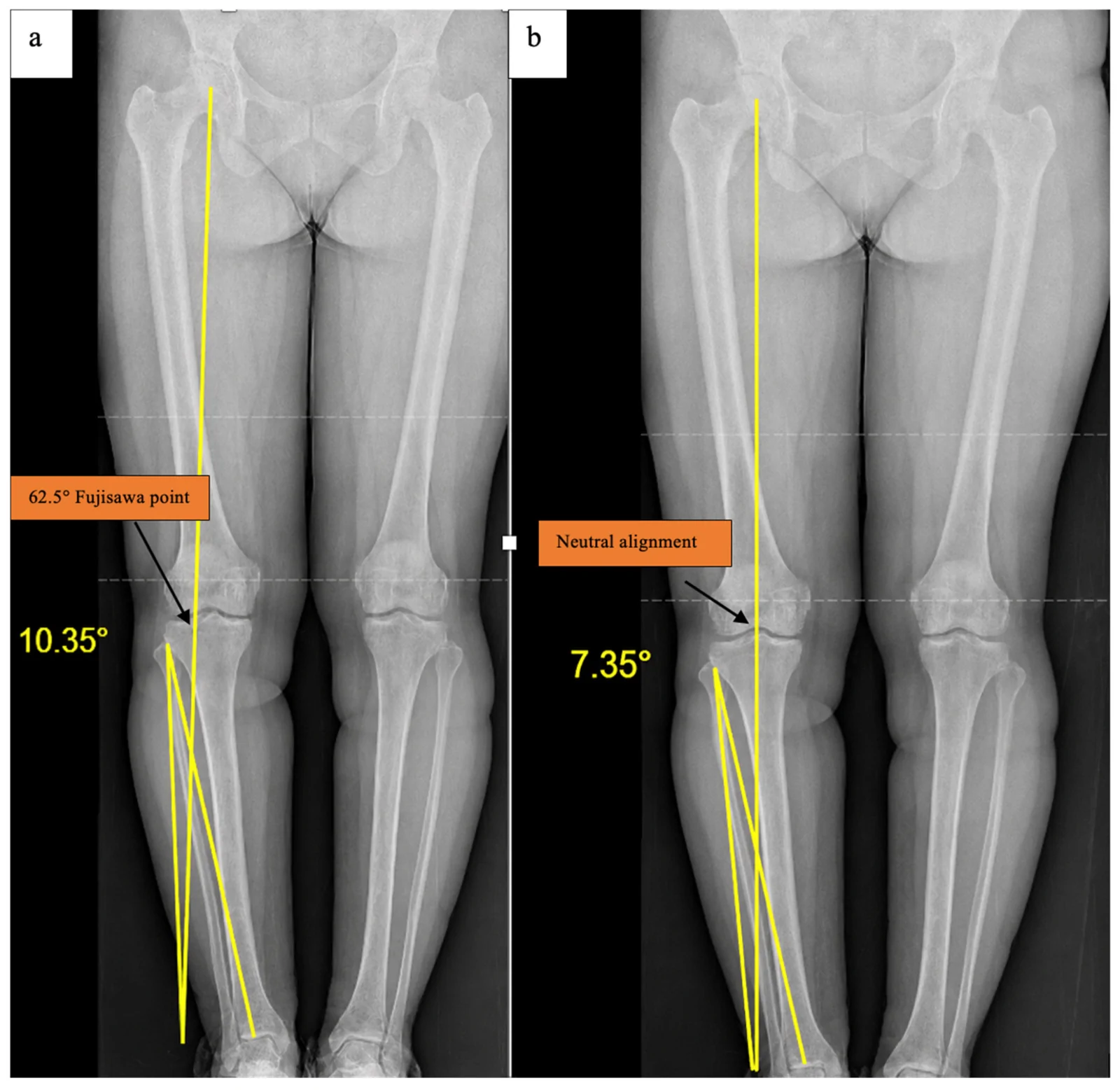
Preoperative mechanical-axis planning using the Miniaci method. (**a**) Planning toward the Fujisawa reference, corresponding to approximately 62.5% of the tibial plateau width; (**b**) planning toward the neutral mechanical axis, corresponding approximately to HKA 0°. The displayed correction angles illustrate the different Miniaci planning requirements for the two reference directions.

**Figure 3:**
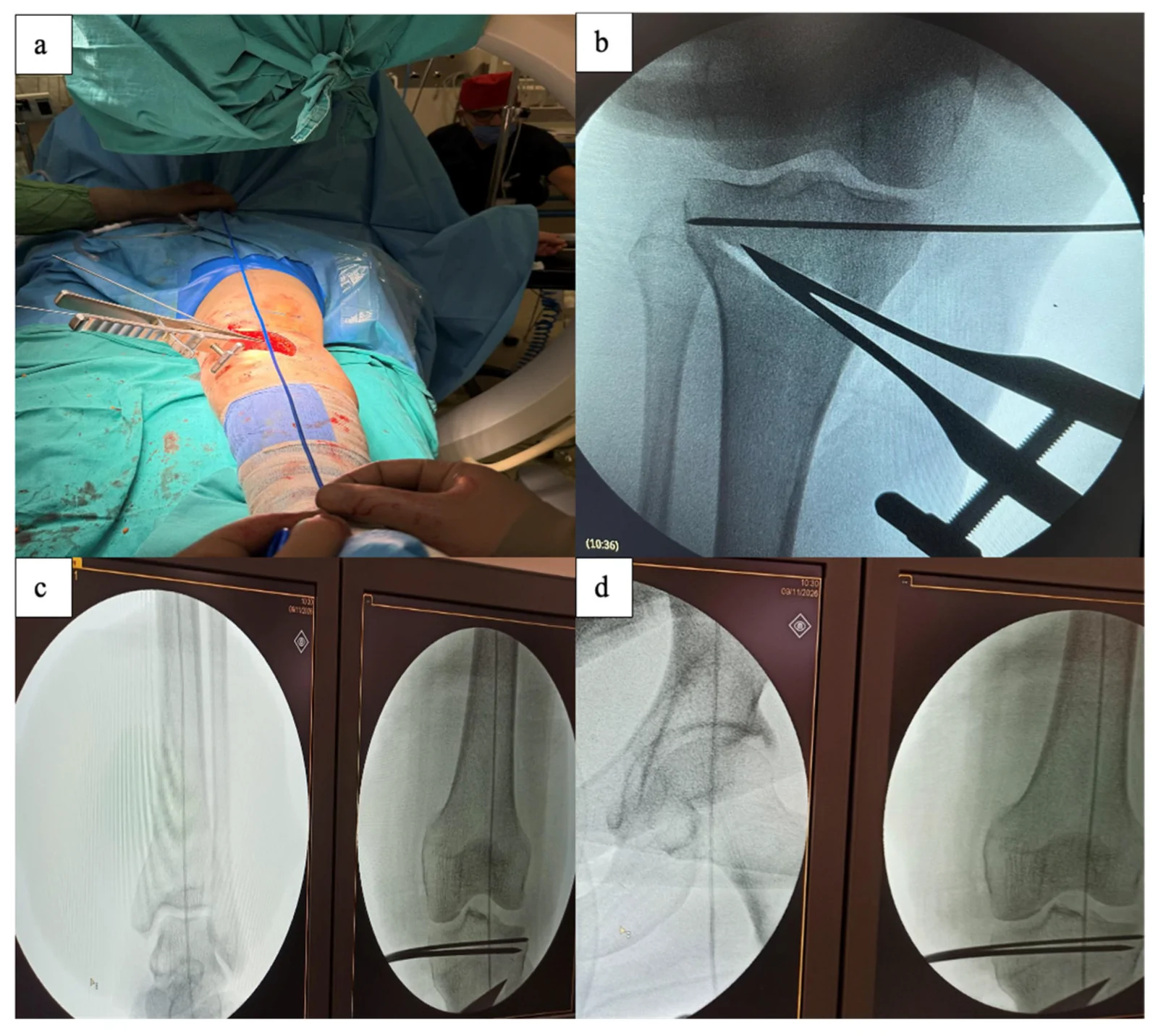
Intraoperative implementation and fluoroscopic verification of the selected mechanical-axis correction direction during medial opening-wedge high tibial osteotomy (MOWHTO). (**a**) Intraoperative assessment of coronal mechanical alignment using the electrocautery-cord cable technique after diagnostic arthroscopy and completion of the correction-score assessment. (**b**) Gradual opening of the osteotomy using a distractor. (**c**) Fluoroscopic confirmation of the distal reference, with the cable centered over the ankle. (**d**) Fluoroscopic confirmation of the proximal reference, with the cable centered over the femoral head; with the knee positioned in a true anteroposterior projection, passage of the mechanical axis across the tibial plateau was assessed relative to the selected neutral/slight-valgus or Fujisawa-oriented reference.

**Figure 4:**
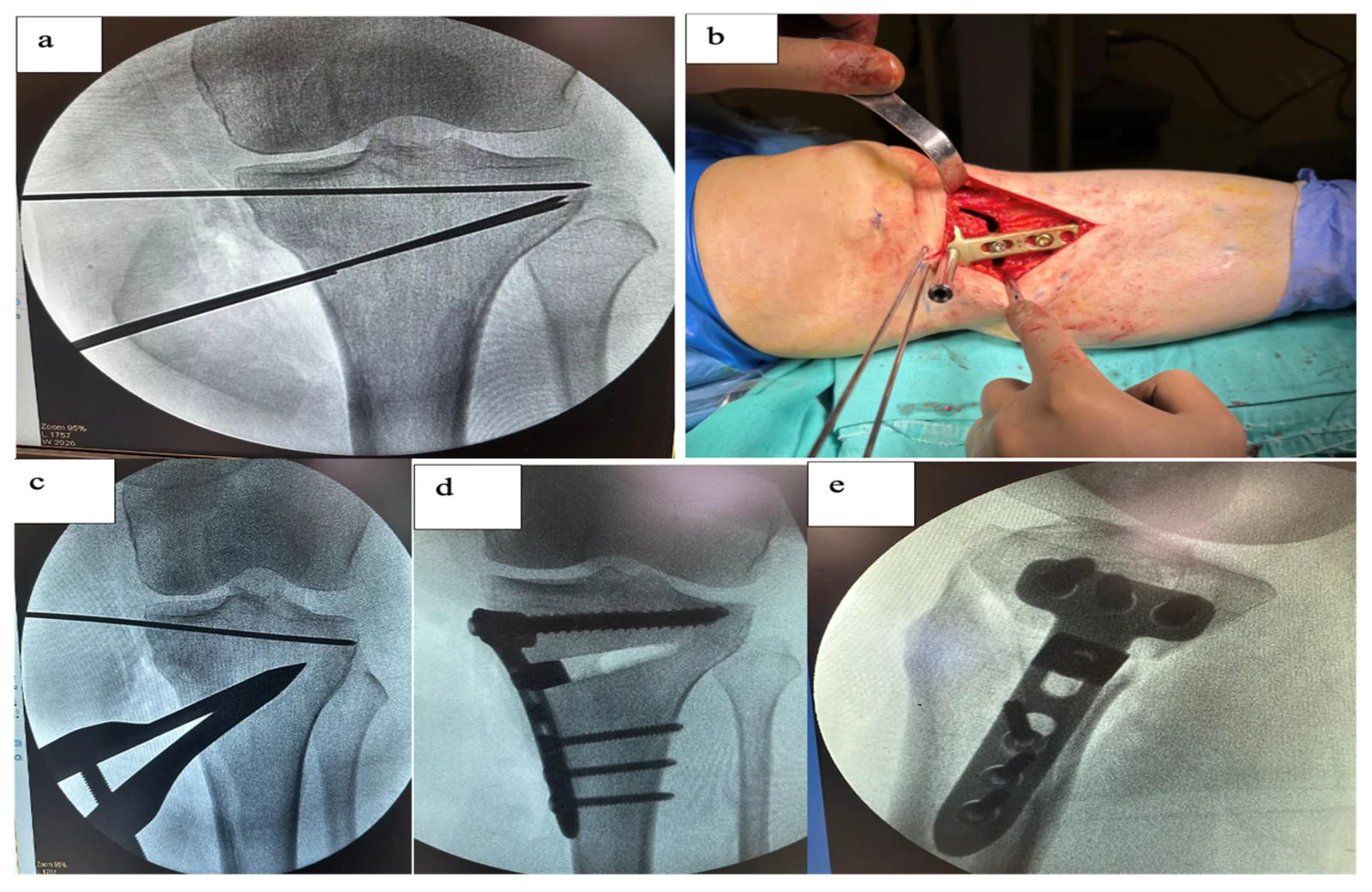
Key intraoperative steps of medial opening-wedge high tibial osteotomy (MOWHTO). (**a**) Placement of protective stop wires and osteotomy-guiding Kirschner wires; (**b**) biplanar osteotomy; (**c**) controlled opening of the osteotomy using a distractor; (**d**) anteroposterior fluoroscopic view of the final fixation; (**e**) lateral fluoroscopic view of the final fixation.

**Figure 5:**
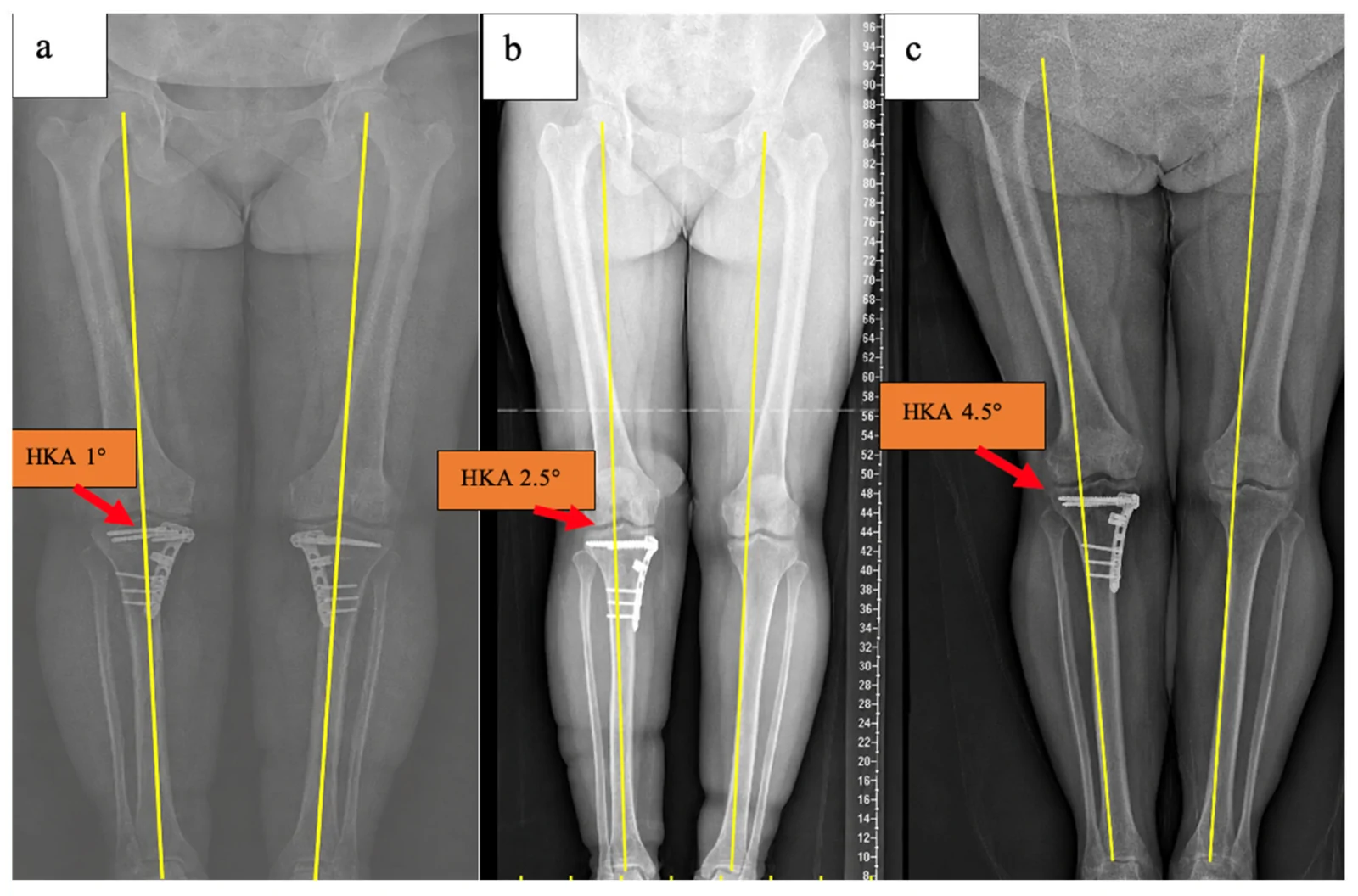
Representative examples of postoperative coronal mechanical alignment at 6 weeks: (**a**) near-neutral/slight valgus (HKA, 1.0°); (**b**) intermediate valgus (HKA, 2.5°); and (**c**) greater valgus (HKA, 4.5°). Mechanical alignment was assessed using the hip–knee–ankle angle (HKA) on standing weight-bearing full-length lower-extremity radiographs.

**Figure 6:**
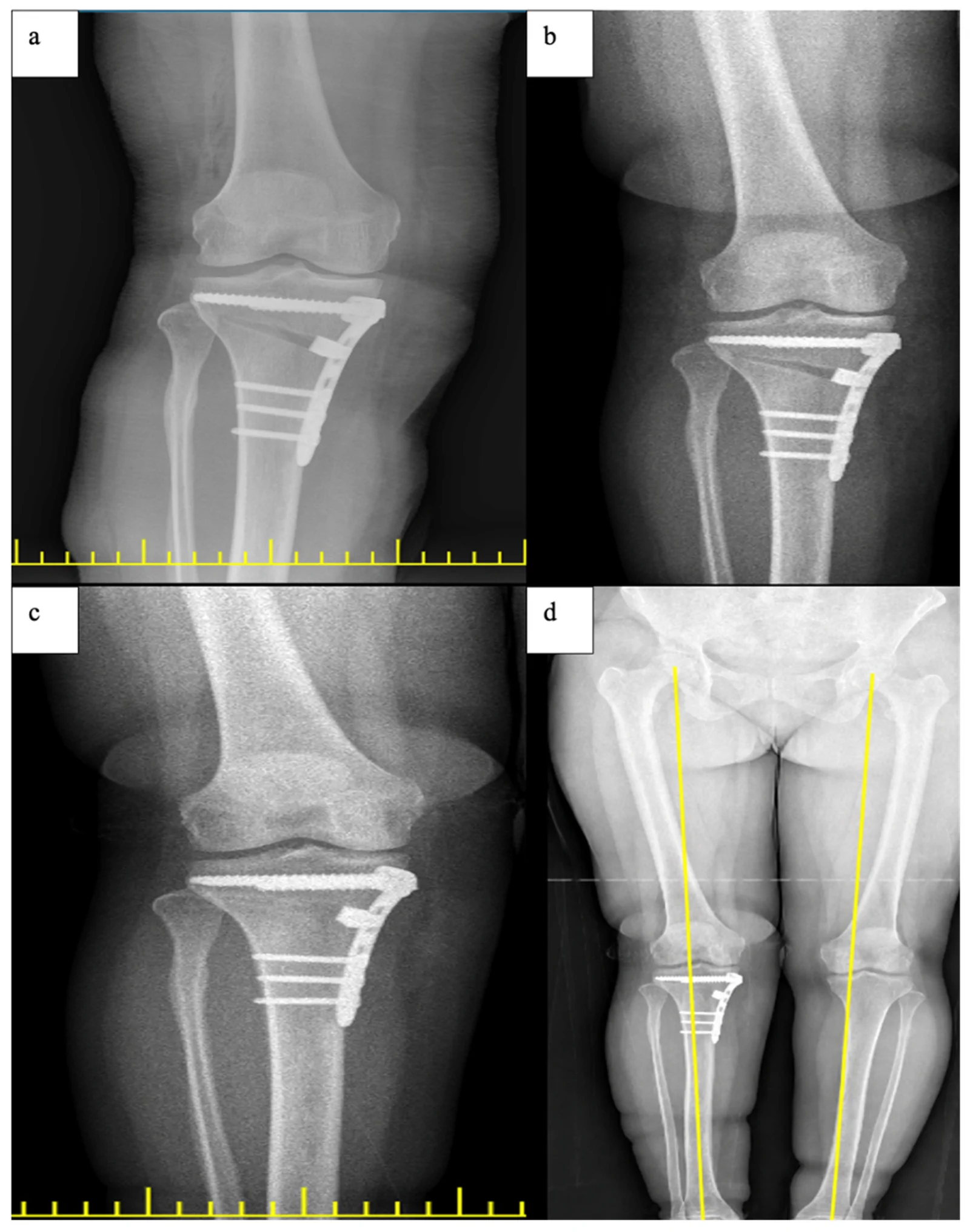
Serial radiographic progression of osteotomy-site healing following medial opening-wedge high tibial osteotomy (MOWHTO). Radiographs obtained from the same patient at (**a**) 2 weeks, (**b**) 6 weeks, (**c**) 3 months, and (**d**) 6 months postoperatively demonstrate progressive trabecular bone bridging across the osteotomy site.

**Table 1:** Individualized correction-direction and selective-grafting algorithm used in the study cohort.

Step	Assessment	Decision
**Preoperative reference planning**	Miniaci method on standing full-length weight-bearing radiographs	Two reference directions defined: neutral mechanical axis (approximately HKA 0°; WBL through the knee center) and Fujisawa reference (approximately 62.5% of tibial plateau width)
**Correction score**	Age < 55 years	1 point
	KL grade 3	1 point
	Medial-compartment ICRS grade 4	1 point
	MMPRT or repairable longitudinal/bucket-handle medial meniscal tear	1 point
**Target-direction selection**	Total score 0–1	Correction directed toward the neutral/slight-valgus reference
	Total score 2–4	Correction directed toward the Fujisawa reference
**Interpretation of score**	Individual scores 0–4	Individual score values were not assigned separate fixed HKA values or WBL percentages; the score was used categorically to select the reference direction
**Osteotomy opening**	Selected mechanical-axis direction and Miniaci planning	Correction performed using the available 10- or 12.5-mm osteotomy opening in the included cohort
**Intraoperative verification**	Fluoroscopic electrocautery-cord cable technique	Mechanical-axis passage across the tibial plateau assessed with the cable centered over the ankle distally and femoral head proximally
**Selective grafting**	Osteotomy gap width and lateral-hinge integrity	No routine grafting for a 10-mm opening with an intact hinge; graft requirement reassessed for a 12.5-mm opening; hinge disruption favored grafting

**Abbreviations:** ICRS, International Cartilage Repair Society; KL, Kellgren–Lawrence; MMPRT, medial meniscus posterior root tear.

**Table 2:** Demographic, clinical, surgical, and radiological characteristics of the study cohort.

Variable	Overall Cohort (n = 188)
Age, years	52.64 ± 4.74
Female, n (%)	128 (68.1)
Male, n (%)	60 (31.9)
BMI, kg/m^2^	29.36 ± 3.06
Follow-up duration, months	25.48 ± 6.80
Preoperative HKA, °	−9.45 ± 2.06
Postoperative 6-week HKA, °	2.88 ± 1.21
Final follow-up HKA, °	2.38 ± 1.32
**Postoperative alignment category, n (%)**	
Near-neutral/slight valgus (HKA <2.0°)	44 (23.4)
Intermediate valgus (HKA 2.0–2.9°)	39 (20.7)
Greater valgus (HKA ≥3.0°)	105 (55.9)
**Kellgren–Lawrence grade, n (%)**	
Grade 2	70 (37.2)
Grade 3	118 (62.8)
**ICRS chondral damage grade, n (%)**	
Grade 2	59 (31.4)
Grade 3	91 (48.4)
Grade 4	38 (20.2)
MMPRT, n (%)	15 (8.0)
Other medial meniscal pathology, n (%)	54 (28.7)
**Intra-articular treatment pattern, n (%)**	
Diagnostic arthroscopy only	119 (63.3)
MMPRT repair	15 (8.0)
Other medial meniscal repair	14 (7.4)
Partial meniscectomy + chondroplasty + microfracture	40 (21.3)
**Osteotomy opening, n (%)**	
10 mm	118 (62.8)
12.5 mm	70 (37.2)
Autologous tricortical iliac crest graft, n (%)	96 (51.1)
Lateral cortical hinge fracture, n (%)	24 (12.8)

**Data are presented as mean ± standard deviation or n (%). Abbreviations:** BMI, body mass index; HKA, hip–knee–ankle angle; ICRS, International Cartilage Repair Society; MMPRT, medial meniscus posterior root tear.

**Table 3:** Clinical outcomes before MOWHTO and at the final follow-up.

Clinical Outcome	Preoperative	Final Follow-Up	Mean Improvement (95% CI)	*p*-Value
**IKDC**	39.37 ± 8.45	80.11 ± 8.26	40.74 ± 11.61 (39.07–42.41)	**<0.001**
**Lysholm**	47.46 ± 8.60	79.26 ± 10.27	31.80 ± 12.71 (29.97–33.63)	**<0.001**
**VAS**	6.99 ± 0.95	1.82 ± 0.40	5.17 ± 1.01 (5.03–5.32)	**<0.001**

**Data are presented as mean ± standard deviation.** Improvements in IKDC and Lysholm scores were calculated as the final follow-up score minus the preoperative score, whereas VAS improvement was calculated as the preoperative score minus the final follow-up score; thus, positive values indicate clinical improvement. The 95% CIs refer to the mean within-patient change. **Abbreviations:** CI, confidence interval; IKDC, International Knee Documentation Committee; MOWHTO, medial opening-wedge high tibial osteotomy; VAS, Visual Analog Scale.

**Table 4:** Multivariable associations between postoperative 6-week HKA and clinical outcomes at final follow-up.

Outcome	β per 1° Increase in Valgus HKA	Robust SE	95% CI	*p*-Value	Adjusted R^2^
**Final follow-up IKDC †**	**1.49**	**0.74**	**0.04–2.93**	**0.044**	**0.050**
**Final follow-up Lysholm**	**2.61**	**0.77**	**1.10–4.13**	**<0.001**	**0.204**
Final follow-up VAS	0.004	0.028	−0.050 to 0.058	0.883	0.030

† The final follow-up IKDC score was designated as the primary clinical outcome for the present analysis. **Model adjustment:** All models were adjusted for age, sex, BMI, follow-up duration, preoperative HKA, KL grade, medial-compartment ICRS grade, MMPRT, other medial meniscal pathology, and the corresponding preoperative clinical score. ICRS grade was modeled as an ordinal variable. Operative variables arising during the treatment pathway, including osteotomy opening size, lateral cortical hinge fracture, and iliac crest graft use, were not included in the primary models. **Statistical note:** β represents the unstandardized regression coefficient associated with each 1° increase in valgus HKA at 6 weeks postoperatively. Standard errors, 95% CIs, and *p*-values were calculated using HC3 heteroskedasticity-robust standard errors. **Abbreviations:** BMI, body mass index; CI, confidence interval; HKA, hip–knee–ankle angle; ICRS, International Cartilage Repair Society; IKDC, International Knee Documentation Committee; KL, Kellgren–Lawrence; MMPRT, medial meniscus posterior root tear; SE, standard error; VAS, Visual Analog Scale.

**Table 5:** Clinical outcomes according to postoperative 6-week HKA categories.

Outcome	Near-Neutral/Slight Valgus HKA < 2.0° (n = 44)	Intermediate Valgus HKA 2.0–2.9° (n = 39)	Greater Valgus HKA ≥ 3.0° (n = 105)	*p*-Value
Final follow-up IKDC	**80.75 [66.05–86.20]**	**81.60 [77.80–85.55]**	**81.60 [77.80–86.20]**	0.125 †
ΔIKDC	38.33 ± 11.95	43.27 ± 12.00	40.81 ± 11.21	0.152 ‡
**Final follow-up Lysholm**	**71.00 [63.02–78.05]**	**83.40 [75.20–89.15]**	**81.90 [78.20–86.80]**	**<0.001 †**
**ΔLysholm**	**27.30 [14.80–36.15]**	**32.70 [21.55–38.40]**	**34.50 [28.50–41.60]**	**<0.001 †**
Final follow-up VAS	**1.75 [1.50–2.10]**	**1.50 [1.50–2.00]**	**1.50 [1.50–2.10]**	0.450 †
VAS improvement	5.08 ± 1.00	5.05 ± 1.10	5.25 ± 0.98	0.464 ‡

Data are presented as mean ± standard deviation for normally distributed variables and as median [interquartile range] for non-normally distributed variables. ΔIKDC and ΔLysholm were calculated as the final follow-up value minus the preoperative value, whereas VAS improvement was calculated as the preoperative value minus the final follow-up value; thus, positive values indicate clinical improvement. † Kruskal–Wallis test. ‡ one-way analysis of variance (ANOVA). For outcomes with a significant overall Kruskal–Wallis test, post-hoc pairwise comparisons were performed with adjustment for multiple comparisons. For final follow-up Lysholm, the near-neutral/slight-valgus group differed significantly from both the intermediate-valgus and greater-valgus groups (Holm-adjusted *p* < 0.001 for both comparisons), whereas the intermediate- and greater-valgus groups did not differ significantly (Holm-adjusted *p* = 0.852). For ΔLysholm, only the comparison between the near-neutral/slight-valgus and greater-valgus groups remained statistically significant after Holm adjustment (*p* < 0.001). Abbreviations: HKA, hip–knee–ankle angle; IKDC, International Knee Documentation Committee; VAS, Visual Analog Scale.

**Table 6:** Maintenance of mechanical correction and radiological outcomes.

Variable	Overall Cohort (n = 188)
Postoperative 6-week HKA, °	2.88 ± 1.21
Final follow-up HKA, °	2.38 ± 1.32
ΔHKA, °†	−0.50 ± 0.28
Postoperative 6-week MPTA, °	88.23 ± 2.15
Final follow-up MPTA, °	87.84 ± 2.16
ΔMPTA, °†	−0.39 ± 0.23
Preoperative PTS, °	8.50 ± 1.74
Postoperative PTS, °	8.70 ± 1.90
ΔPTS, °‡	0.20 ± 0.80
Time to first documented union, weeks	12.2 (10.0–17.8)
Delayed union, n (%)	0 (0.0)
Nonunion, n (%)	0 (0.0)

**Data are presented as mean ± standard deviation, median (range), or n (%), as appropriate.** ° indicates degrees; Δ indicates change; † ΔHKA and ΔMPTA were calculated as final follow-up − postoperative 6-week values; negative values indicate a decrease during follow-up. ‡ ΔPTS was calculated as postoperative − preoperative value. Abbreviations: HKA, hip–knee–ankle angle; MPTA, medial proximal tibial angle; PTS, posterior tibial slope.

**Table 7:** Secondary unadjusted comparisons according to graft use.

Variable	Grafted (n = 96)	Nongrafted (n = 92)	*p*-Value
Time to first documented union, weeks	12.8 [11.55–13.30]	12.0 [12.00–13.60]	0.863 §
ΔHKA, °†	−0.49 ± 0.27	−0.51 ± 0.28	0.529
ΔMPTA, °†	−0.40 ± 0.22	−0.37 ± 0.23	0.345

**Data are presented as mean ± standard deviation or median [interquartile range], as appropriate.** ° indicates degrees; Δ indicates change; † ΔHKA and ΔMPTA were calculated as final follow-up − postoperative 6-week values; negative values indicate a decrease during follow-up. § Mann–Whitney U test. The graft-use comparisons were secondary unadjusted analyses and should not be interpreted as estimates of the causal effect of grafting. Abbreviations: HKA, hip–knee–ankle angle; MPTA, medial proximal tibial angle.

**Table 8:** Perioperative and postoperative complications.

Complication	Overall Cohort (n = 188)
Lateral cortical hinge fracture, n (%)	24 (12.8)
Type 1	17 (9.0)
Type 2	5 (2.7)
Type 3	2 (1.1)
Prolonged wound drainage (>3 postoperative days), n (%)	14 (7.4)
Culture-negative prolonged wound drainage	10 (5.3)
Culture-positive postoperative infection	4 (2.1)
MRSA-positive infection	4 (2.1)
Surgical debridement for infection, n (%)	4 (2.1)
Implant removal for infection, n (%)	0 (0.0)
Delayed union, n (%)	0 (0.0)
Nonunion, n (%)	0 (0.0)
Mechanical revision, n (%)	0 (0.0)

**Data are presented as n (%). Abbreviation:** MRSA, methicillin-resistant *Staphylococcus aureus*.

## Data Availability

The data supporting the findings of this study are available from the corresponding author upon reasonable request.

## Supplementary Materials

The following supporting information can be downloaded at: https://www.mdpi.com/article/10.3390/jcm15208110/s1, Table S1: Interobserver reliability of radiographic measurements; Table S2: Full multivariable linear regression models evaluating the association between 6-week postoperative HKA and final IKDC, Lysholm, and VAS scores.

## References

[1] Pérez López C.J., Rivera Troia F.M., Ramírez N. (2025). Clinical outcomes following medial opening wedge high tibial osteotomy in patients with medial compartment grade 4 chondromalacia. J. Am. Acad. Orthop. Surg. Glob. Res. Rev..

[2] Arai Y., Nakagawa S., Inoue A., Fujii Y., Cha R., Nakamura K., Takahashi K. (2025). Long-term outcomes and prognostic factors of medial open wedge high tibial osteotomy for medial compartment knee osteoarthritis or osteonecrosis. J. Clin. Med..

[3] Ghasemi S.A., Murray B.C., Buksbaum J.R., Shin J., Fragomen A., Rozbruch S.R. (2022). Opening wedge high tibial osteotomy for medial compartment knee osteoarthritis: Planning and improving outcomes: Case series and literature review. J. Clin. Orthop. Trauma.

[4] Murray R., Winkler P.W., Shaikh H.S., Musahl V. (2021). High tibial osteotomy for varus deformity of the knee. J. Am. Acad. Orthop. Surg. Glob. Res. Rev..

[5] Kobayashi H., Saito S., Akamatsu Y., Kumagai K., Nejima S., Inaba Y. (2022). The relationship between the “Fujisawa point” and anatomical femorotibial angle following simulated open wedge high tibial osteotomy. BMC Musculoskelet. Disord..

[6] Kang B.Y., Lee D.K., Kim H.S., Wang J.H. (2022). How to achieve an optimal alignment in medial opening wedge high tibial osteotomy?. Knee Surg. Relat. Res..

[7] Atkinson H.F., Birmingham T.B., Schulz J.M., Primeau C.A., Leitch K.M., Pritchett S.L., Holdsworth D.W., Giffin J.R. (2022). High tibial osteotomy to neutral alignment improves medial knee articular cartilage composition. Knee Surg. Sports Traumatol. Arthrosc..

[8] Lee S.S., Kim J.H., Kim S., Jung E.Y., Ryu D.J., Lee D.K., Wang J.H. (2022). Avoiding overcorrection to increase patient satisfaction after open wedge high tibial osteotomy. Am. J. Sports Med..

[9] Yoon T.H., Choi C.H., Kim S.J., Kim S.H., Kim N.H., Jung M. (2019). Effect of medial open-wedge high tibial osteotomy on the patellofemoral joint according to postoperative realignment. Am. J. Sports Med..

[10] Ishibashi K., Tsushima T., Sasaki E., Ishibashi H.K., Sakamoto Y., Kimura Y., Ishibashi Y. (2026). Individual target alignment for opening-wedge high tibial osteotomy with reference to the arithmetic hip-knee-ankle angle. J. Orthop..

[11] Kim S.E., Kwak J., Ro D.H., Lee M.C., Han H.S. (2024). The use of medial joint opening rather than mechanical axis deviation to determine the clinical outcomes after high tibial osteotomy. Bone Jt. J..

[12] Blackburn J., Ansari F., Porteous A., Murray J. (2022). The relationships between coronal plane alignments and patient-reported outcomes following high tibial osteotomy: A systematic review. Cartilage.

[13] Oktem U., Dastan M.C., Avci H., Bulut M., Ozaltin G.E., Ocguder D.A., Tecimel O., Bingol I. (2026). High tibial osteotomy is associated with improvements in both knee and ankle alignment in medial compartment osteoarthritis. J. Clin. Med..

[14] Miniaci A., Ballmer F.T., Ballmer P.M., Jakob R.P. (1989). Proximal tibial osteotomy. A new fixation device. Clin. Orthop. Relat. Res..

[15] Schröter S., Elson D.W., Ateschrang A., Ihle C., Stöckle U., Dickschas J., Harrer J. (2017). Lower limb deformity analysis and the planning of an osteotomy. J. Knee Surg..

[16] Fujisawa Y., Masuhara K., Shiomi S. (1979). The effect of high tibial osteotomy on osteoarthritis of the knee. An arthroscopic study of 54 knee joints. Orthop. Clin. North Am..

[17] Lee D.C., Byun S.J. (2019). High tibial osteotomy for knee osteoarthritis: The proper correction of the mechanical axis. J. Orthop. Surg. Res..

[18] Lee S.J., Kim J.H., Baek E., Ryu H.S., Han D., Choi W. (2021). Incidence and factors affecting the occurrence of lateral hinge fracture after medial opening-wedge high tibial osteotomy. Orthop. J. Sports Med..

[19] Mao Y., Yao L., Li J., Li J., Xiong Y. (2024). No superior bone union outcomes with allografts compared to no grafts and autografts following medial opening wedge high tibial osteotomy: A retrospective cohort study. Orthop. Surg..

[20] Ruzbarsky J.J., Arner J.W., Dornan G.J., Provencher M.T., Vidal A.F. (2021). Tibial slope can be maintained during medial opening-wedge proximal tibial osteotomy with sagittally oriented hinge, posterior plate position, and knee hyperextension: A cadaveric study. Arthroscopy.

[21] Celik D., Coşkunsu D., Kılıçoğlu Ö., Ergönül Ö., Irrgang J.J. (2014). Translation and cross-cultural adaptation of the International Knee Documentation Committee Subjective Knee Form into Turkish. J. Orthop. Sports Phys. Ther..

[22] Celik D., Coşkunsu D., Kılıçoğlu Ö. (2013). Translation and cultural adaptation of the Turkish Lysholm Knee Scale: Ease of use, validity, and reliability. Clin. Orthop. Relat. Res..

[23] Ahrend M.D., Petzold D., Histing T., Ihle C., Schröter S., Herbst M. (2025). High survival rates and patient satisfaction 12 years after medial open wedge high tibial osteotomy surgery: A prospective cohort study. Knee Surg. Sports Traumatol. Arthrosc..

[24] van Houten A.H., Heesterbeek P.J.C., van Heerwaarden R.J., van Tienen T.G., Wymenga A.B. (2014). Medial open wedge high tibial osteotomy: Can delayed or nonunion be predicted?. Clin. Orthop. Relat. Res..

[25] Song S.J., Yoon K.H., Lee S.J., Park C.H. (2024). Impact of a unilateral high tibial osteotomy with a large correction on functional lateral acetabular coverage of the hip joint. Orthop. J. Sports Med..

[26] Long J.S., Ervin L.H. (2000). Using heteroscedasticity consistent standard errors in the linear regression model. Am. Stat..

[27] Yin Y., Zhang Y., Li S., Hou Z., Guo J., Zhang R. (2020). What is the relationship between the “Fujisawa point” and postoperative knee valgus angle? A theoretical, computer-based study. Knee.

[28] Patel S., Haunschild E., Gilat R., Knapik D., Evuarherhe A., Parvaresh K.C., Chahla J., Yanke A.B., Cole B.J. (2021). Defining clinically significant outcomes following high tibial osteotomy with or without concomitant procedures. J. Cartil. Jt. Preserv..

[29] Murphy M.C., Mosler A.B., Rio E.K., Coventry M., Raj I.S., Chivers P.T., Arendt-Nielsen L., Alfieri F.M., Bjurström M.F., Larsen D.B. (2025). Can quantitative sensory testing predict treatment outcomes in hip and knee osteoarthritis? A systematic review and meta-analysis of individual participant data. Pain.

[30] Sánchez Romero E.A., Fernández Carnero J., Villafañe J.H., Calvo-Lobo C., Ochoa Sáez V., Burgos Caballero V., Laguarta Val S., Pedersini P., Pecos Martín D. (2020). Prevalence of myofascial trigger points in patients with mild to moderate painful knee osteoarthritis: A secondary analysis. J. Clin. Med..

[31] Kim J.H., Lee D.K., Park Y.B. (2021). Computed tomography detects hinge fractures after medial opening wedge high tibial osteotomy: A systematic review. Arthroscopy.

[32] Bei T., Yang L., Huang Q., Wu J., Liu J. (2022). Effectiveness of bone substitute materials in opening wedge high tibial osteotomy: A systematic review and meta-analysis. Ann. Med..

[33] Mao Y., You M., Zhang L., Li J., Fu W. (2023). Lack of efficacy of bone void filling materials in medial opening-wedge high tibial osteotomy: A systematic review and network meta-analysis. Arthroscopy.

[34] Lin L., Jiang S., Yang S., Yang G., Xie B., Zhang L. (2024). Identical clinical outcomes between neutral and classic targeted alignments after high tibial osteotomy in medial meniscus posterior root tear: A prospective randomized study. Int. Orthop..

[35] Erdem A., Uçan V., Lebe G., Geçkalan M.A., Demirkıran C.B., Toprak A., Elmalı N. (2025). Impact of mechanical axis position and coronal plane alignment phenotypes on clinical outcomes in medial opening wedge high tibial osteotomy. Int. Orthop..

