# Supplementary material for: Association Between Postoperative Mechanical Alignment and Clinical Outcomes After Medial Opening-Wedge High Tibial Osteotomy: A Retrospective Cohort Study
Source: J Clin Med. 2026 Oct 19;15(20):8110. doi: 10.3390/jcm15208110 (PMC13706026; doi:10.3390/jcm15208110)
Supplement: Supplementary file 1 [file jcm-15-08110-s001.zip › jcm-4545792-supplementary.pdf]

Supplementary Table S1. Interobserver reliability of radiographic measurements.

Radiographic measurement	n	ICC	95% CI	Reliability
**Preoperative HKA**	**188**	**0.999**	**0.998–0.999**	**Excellent**
**6-week postoperative HKA**	**188**	**0.999**	**0.999–1.000**	**Excellent**
**Final follow-up HKA**	**188**	**0.999**	**0.999–1.000**	**Excellent**
**Preoperative MPTA**	**188**	**1.000**	**1.000–1.000**	**Excellent**
**6-week postoperative MPTA**	**188**	**1.000**	**1.000–1.000**	**Excellent**
**Final follow-up MPTA**	**188**	**1.000**	**1.000–1.000**	**Excellent**
**Preoperative LDFA**	**188**	**0.696**	**0.586–0.790**	**Moderate**
**6-week postoperative LDFA**	**188**	**0.651**	**0.557–0.736**	**Moderate**
**Final follow-up LDFA**	**188**	**0.422**	**0.306–0.524**	**Poor**
**Preoperative PTS**	**188**	**0.994**	**0.988–0.998**	**Excellent**
**Postoperative PTS**	**188**	**0.980**	**0.969–0.989**	**Excellent**

Table footnote:

ICC, intraclass correlation coefficient; CI, confidence interval; HKA, hip–knee–ankle angle; MPTA, medial proximal tibial angle; LDFA, lateral distal femoral angle; PTS, posterior tibial slope. ICCs were calculated using a two-way random-effects model with an absolute-agreement definition based on the independent measurements of two observers in the entire cohort (n = 188). Ninety-five percent CIs were obtained using 10,000 bootstrap resamples. ICC values were interpreted as <0.50, poor; 0.50–0.75, moderate; 0.75–0.90, good; and >0.90, excellent reliability.

Supplementary Table S2. Complete multivariable regression models for the associations between postoperative 6-week HKA and final follow-up clinical outcomes

Predictor	IKDC β (95% CI)	p	Lysholm β (95% CI)	p	VAS β (95% CI)	p
**Postoperative 6-week HKA, per 1° valgus**	**1.49 (0.04 to 2.93)**	**0.044**	**2.61 (1.10 to 4.13)**	**<0.001**	**0.004 (−0.050 to 0.058)**	**0.883**
Age, years	−0.15 (−0.44 to 0.13)	0.296	0.08 (−0.21 to 0.36)	0.599	0.006 (−0.008 to 0.020)	0.386
Male sex	−0.84 (−3.73 to 2.06)	0.571	−0.80 (−3.90 to 2.31)	0.614	0.069 (−0.063 to 0.201)	0.303
BMI, kg/m²	−0.38 (−0.75 to −0.02)	0.038	0.12 (−0.31 to 0.55)	0.579	0.014 (−0.004 to 0.032)	0.131
Follow-up duration, months	0.02 (−0.15 to 0.20)	0.779	−0.01 (−0.22 to 0.21)	0.954	0.002 (−0.007 to 0.011)	0.679
Preoperative HKA, per 1°	0.56 (−0.07 to 1.20)	0.084	0.17 (−0.55 to 0.90)	0.640	−0.030 (−0.071 to 0.011)	0.155
KL grade 3†	−0.31 (−2.85 to 2.23)	0.811	2.04 (−0.90 to 4.97)	0.173	0.051 (−0.077 to 0.178)	0.436
ICRS grade, per 1-grade increase	−3.01 (−5.78 to −0.24)	0.033	0.97 (−1.83 to 3.78)	0.496	0.159 (0.030 to 0.288)	0.016
MMPRT	−1.33 (−8.17 to 5.50)	0.702	−8.34 (−15.51 to −1.17)	0.023	0.082 (−0.183 to 0.348)	0.544

Other medial meniscal pathology	2.36 (−2.24 to 6.96)	0.315	−3.57 (−8.18 to 1.04)	0.129	−0.060 (−0.263 to 0.144)	0.566
Corresponding preoperative clinical score‡	0.006 (−0.137 to 0.149)	0.934	0.096 (−0.076 to 0.267)	0.274	0.016 (−0.055 to 0.086)	0.659

Values are unstandardized regression coefficients (β) with 95% confidence intervals. Standard errors, confidence intervals, and p-values were calculated using HC3 heteroskedasticity-robust standard errors. Each outcome was analyzed in a separate multivariable linear regression model. The primary models included postoperative 6-week HKA together with baseline/pre-exposure covariates: age, sex, BMI, follow-up duration, preoperative HKA, KL grade, ICRS grade, MMPRT, other medial meniscal pathology, and the corresponding preoperative clinical score. Operative variables arising during the treatment pathway, including osteotomy opening size, lateral cortical hinge fracture, and iliac crest graft use, were not included in the primary models.

† **Reference category:** KL grade 2.

‡ **The corresponding baseline clinical score was included in each outcome-specific model:** preoperative IKDC for final IKDC, preoperative Lysholm for final Lysholm, and preoperative VAS for final VAS.

For binary variables, the reference categories were female sex, absence of MMPRT, and absence of other medial meniscal pathology. ICRS grade was modeled as an ordinal variable, with the coefficient representing the estimated change in outcome per one-grade increase.

Abbreviations: BMI, body mass index; CI, confidence interval; HKA, hip–knee–ankle angle; ICRS, International Cartilage Repair Society; IKDC, International Knee Documentation Committee; KL, Kellgren–Lawrence; MMPRT, medial meniscus posterior root tear; VAS, Visual Analog Scale.
